# Cardiorenal effectiveness of empagliflozin vs. glucagon-like peptide-1 receptor agonists: final-year results from the EMPRISE study

**DOI:** 10.1186/s12933-024-02150-0

**Published:** 2024-02-08

**Authors:** Phyo T. Htoo, Helen Tesfaye, Sebastian Schneeweiss, Deborah J. Wexler, Brendan M. Everett, Robert J. Glynn, Niklas Schmedt, Lisette Koeneman, Anouk Déruaz-Luyet, Julie M. Paik, Elisabetta Patorno

**Affiliations:** 1 Division of Pharmacoepidemiology and Pharmacoeconomics, Department of Medicine, Brigham and Women’s Hospital and Harvard Medical School, 1620 Tremont Street, Suite 3030, Boston, MA 02120 USA; 2 Massachusetts General Hospital Diabetes Center, Harvard Medical School, Boston, USA; 3 Divisions of Cardiovascular and Preventive Medicine, Department of Medicine, Brigham and Women’s Hospital, Harvard Medical School, 75 Francis Street, Boston, MA USA; 4 Global Epidemiology, Boehringer Ingelheim International GmbH (Germany) DE, Berlin, Germany; 5 Global Medical Affairs, Lilly Deutschland GmbH, Bad Homburg, Germany; 6 Division of Renal (Kidney) Medicine, Brigham and Women’s Hospital, Boston, MA USA

**Keywords:** Empagliflozin, SGLT2i, GLP-1RA, Cardiovascular disease, Type 2 diabetes, Chronic kidney disease, Effectiveness

## Abstract

**Background:**

No randomized clinical trials have directly compared the cardiorenal effectiveness of empagliflozin and GLP-1RA agents with demonstrated cardioprotective effects in patients with a broad spectrum of cardiovascular risk. We reported the final-year results of the EMPRISE study, a monitoring program designed to evaluate the cardiorenal effectiveness of empagliflozin across broad patient subgroups.

**Methods:**

We identified patients ≥ 18 years old with type 2 diabetes who initiated empagliflozin or GLP-1RA from 2014 to 2019 using US Medicare and commercial claims databases. After 1:1 propensity score matching using 143 baseline characteristics, we evaluated risks of outcomes including myocardial infarction (MI) or stroke, hospitalization for heart failure (HHF), major adverse cardiovascular events (MACE – MI, stroke, or cardiovascular mortality), a composite of HHF or cardiovascular mortality, and progression to end-stage kidney disease (ESKD) (in patients with chronic kidney disease stages 3–4). We estimated hazard ratios (HR) and rate differences (RD) per 1,000 person-years, overall and within subgroups of age, sex, baseline atherosclerotic cardiovascular disease (ASCVD), and heart failure (HF).

**Results:**

We identified 141,541 matched pairs. Compared with GLP-1RA, empagliflozin was associated with similar risks of MI or stroke [HR: 0.99 (0.92, 1.07); RD: -0.23 (-1.25, 0.79)], and lower risks of HHF [HR: 0.50 (0.44, 0.56); RD: -2.28 (-2.98, -1.59)], MACE [HR: 0.90 (0.82, 0.99); RD: -2.54 (-4.76, -0.32)], cardiovascular mortality or HHF [HR: 0.77 (0.69, 0.86); RD: -4.11 (-5.95, -2.29)], and ESKD [0.75 (0.60, 0.94); RD: -6.77 (-11.97, -1.61)]. Absolute risk reductions were larger in older patients and in those with baseline ASCVD/HF. They did not differ by sex.

**Conclusions:**

The cardiovascular benefits of empagliflozin vs. cardioprotective GLP-1RA agents were larger in older patients and in patients with history of ASCVD or HF, while they did not differ by sex. In patients with advanced CKD, empagliflozin was associated with risk reductions of progression to ESKD.

**Supplementary Information:**

The online version contains supplementary material available at 10.1186/s12933-024-02150-0.

## Background

In patients with type 2 diabetes (T2D) and established cardiovascular diseases (CVD), treatment with empagliflozin, a sodium-glucose cotransporter 2 inhibitor (SGLT2i), has demonstrated reductions in the risks of major adverse cardiovascular events (MACE), mortality, hospitalization for heart failure (HHF), and kidney-related outcomes relative to placebo [1]. Glucagon-like peptide-1 receptor agonists (GLP-1RA) have also demonstrated efficacy against MACE and kidney-related outcomes relative to placebo in patients with T2D with or at risk for atherosclerotic CVD (ASCVD) [2–4].

The demonstrated benefits of empagliflozin and GLP-1RA in placebo-controlled trials raise the question of how their benefits compare in broader populations of patients with T2D. To date, no large cardiovascular outcome trials have directly compared empagliflozin vs. GLP-1RA to help guide treatment prescribing. Previous observational studies that compared the effectiveness of SGLT2i and GLP-1RA included only a small number or proportion of patients on empagliflozin [5–10], are based on a single healthcare setting with limited generalizability [9, 11–15], or are too small to evaluate CVD outcomes with reasonable precision [8, 11, 12, 14–18]. Since not all SGLT2i and GLP-1RA agents demonstrated cardiorenal benefits in placebo-controlled trials, prior evidence may not apply to the effectiveness of empagliflozin relative to GLP-1RA agents. Only two studies have compared empagliflozin with GLP-1RA although they focused on older patients only [19] or did not characterize individual cardiovascular outcomes [20]. They also did not evaluate the cardiovascular mortality and kidney outcomes, which are approved indications for empagliflozin [19, 20].

EMPagliflozin compaRative effectIveness and SafEty (EMPRISE) study is a sequentially built population-based monitoring program designed to evaluate the effectiveness and safety of empagliflozin [21]. We present the final-year results from the EMPRISE study comparing the cardiorenal effectiveness of empagliflozin vs. GLP-1RA (restricted to agents with demonstrated cardioprotective effects) across broad population subgroups.

## Methods

We conducted an active-comparator, new-user cohort study [22] using three data sources: two US commercial claims (Optum’s de-identified Clinformatics® Data Mart Database and IBM Marketscan), and Medicare federal insurance data. These databases contain deidentified, longitudinal patient-level data on all reimbursed medical services, including inpatient and outpatient diagnoses and procedures, along with pharmacy dispensing records. The study protocol [EnCEPP (EUPAS20677) and ClinicalTrials.gov (NCT03363464)] was approved by the Institutional Review Board at Mass General Brigham. Data use agreements were in place.

### Study population and exposure assessment

The study population included patients with T2D aged 18 years or older (65 years or older in Medicare), who initiated empagliflozin or cardioprotective GLP-1RA (liraglutide, albiglutide, dulaglutide, injection semaglutide) from 8/2014 to 9/2019 (Additional file 1: sFigure 1). Exenatide and lixisenatide were not considered in the GLP-1RA group due to the lack of demonstrated cardiovascular benefits, and oral semaglutide was not yet approved during the timeframe of this study. Cohort entry was on the date of initiation of empagliflozin or a comparator without history of any SGLT2i or GLP-1RA prescriptions for 12 months, which was defined as the baseline period. We required patients to have continuous coverage for insurance plans and a recorded diagnosis of T2D during this baseline period. We excluded patients with recorded diagnoses of type 1 or secondary diabetes, malignancy, ESKD or kidney replacement therapy, human immunodeficiency virus, solid organ transplant, or a nursing home admission during baseline (Additional file 1: sTable 1). Patients who initiated both empagliflozin and GLP-1RA on the cohort entry date were excluded.

To identify study outcomes, patients were followed from one day after cohort entry until the earliest of: discontinuation of the index drug, switching to the comparator drug class, switching from an initial to an alternative agent within the same class, gap in insurance coverage (> 30 days), death, or end of the study (September 30, 2019). We considered patients to be on medications until 60 days after the end of the last prescription’s supply.

### Outcome definitions

Primary outcomes were (i) a composite of myocardial infarction (MI) or stroke, (ii) hospitalization for heart failure (HHF), defined as an HF diagnosis in the primary discharge position, (iii) MACE, defined as a composite of MI, stroke, or cardiovascular mortality, and (iv) a composite of cardiovascular mortality or HHF. Secondary outcomes included the composite of all primary outcomes (MACE or HHF) (Medicare data only), the composite of MI, stroke, or HHF, HHF defined as diagnosis in all discharge positions (HHF-broad), individual components of MACE, unstable angina, coronary revascularization, all-cause mortality, and ESKD. To allow sufficient time for patients to progress to ESKD, we restricted the population to patients with chronic kidney disease (CKD) stages 3–4, defined using a validated claims-based algorithm [23]. We report detailed outcome definitions in Additional file 1: sTable 2.

We defined primary outcomes using validated claims-based algorithms, with high specificity (93–98%) and positive predictive value (PPV > 98%) [24–26]. Date of death was ascertained from the Vital Status files, with linkage to the Social Security Administration (SSA) data, which has been validated and captures > 95% of deaths in older adults aged > 65 years in the US [27, 28]. Cause of death was ascertained through linkage with the National Death Index data considering only diagnoses in the primary position, and was available only in Medicare data [29].

### Patient characteristics

We identified 143 covariates *a priori* based on literature review and clinical knowledge: demographics, census region, calendar time of cohort entry, modified Charlson/Elixhauser combined comorbidity score [30], validated claims-based frailty index [31], diabetes complications, glucose-lowering medication use on cohort entry and during baseline, cardiovascular diseases, systemic comorbidities, chronic disease medications, and measures of healthcare utilization across different healthcare settings as a proxy for the general health status and the intensity of care. Patient characteristics were measured during the baseline period using administrative enrollment data, diagnosis or procedure codes, and pharmacy National Drug Codes. Data on laboratory results were available in Clinformatics® (~ 45%) and MarketScan (~ 5–10%). We provided the complete list of covariates in Additional file 1: sTable 3.

### Statistical analyses

To improve comparability of the two treatment groups, we 1:1 matched patients initiating empagliflozin with those initiating GLP-1RA based on the estimated propensity score (PS). We estimated PS as the predicted probability of initiating empagliflozin relative to cardioprotective GLP-1RA, separately within each database, after controlling for 143 baseline characteristics using multivariable logistic regression [32]. Since laboratory test results were only available in a subset of the population, they were not included in the PS model. We matched using the nearest neighbor approach without replacement [33], and the maximum allowed difference (caliper) in PS between empagliflozin and GLP-1RA was 0.01 [33]. Balance in covariates, including that of laboratory results, was assessed using absolute standardized mean differences (SMD) (lower values indicate better balance) [34] and the post-matched c-statistic of the model predicting the exposure conditional on baseline covariates (values closer to 0.5 indicate better balance) [35].

To allow tight control of baseline CVD, and risk factors related to evolving treatment indications over time, we conducted PS estimation and matching separately within each baseline CVD subgroup [ASCVD or HF (yes/no)] and each calendar time block (before and after 2018), across a total of 4 strata, within each database. The year 2018 was chosen to better capture the period before and after the shift in treatment guideline recommendations for SGLT2i and GLP-1RA [36–38]. After matching, we pooled all the PS-matched databases, and estimated treatment effects in the final pooled database using stratified likelihood [39]. We estimated hazard ratios (HR) using Cox proportional hazards models, and rate differences (RD) using Mantel-Haenszel methods [39]. We present the cumulative risk of outcomes over the follow-up period using Cumulative Incidence Function (CIF) plots using the Kaplan-Meier method.

We estimated treatment effects within the following subgroups: (i) age (≥ vs. <65 years), (ii) sex (male vs. female), (iii) history of baseline ASCVD (defined as a diagnosis for any major ASCVD, including MI, angina, coronary atherosclerosis or other forms of chronic ischemic heart disease, coronary procedure, ischemic stroke, peripheral arterial disease or surgery, or lower extremity amputation), and (iv) baseline HF. Within each subgroup, the PS was re-estimated, and patients were re-matched on the newly estimated PS. The heterogeneity of estimates across the subgroups was evaluated using the Wald test for homogeneity [39].

### Sensitivity analyses

We undertook several steps to mitigate the potential for unmeasured confounding. First, to reduce unmeasured confounding by kidney function, we restricted the study cohort to patients with at least two dispensed prescriptions for metformin (recommended first line therapy for patients without severely compromised kidney function) during the 6 months prior to cohort entry [40], and without any insulin prescriptions during the baseline period. Second, we restricted the study population to patients with laboratory results data available [i.e., patients with non-missing hemoglobin A1c (HbA1c) and estimated glomerular filtration rate (eGFR)] in Clinformatics® or Marketscan, and matching was re-performed using claims-based variables, HbA1c, and eGFR. Third, we performed 1:1 high-dimensional PS matching, which enriched the original PS with 100 additional empirically identified covariates, based on thousands of candidate covariates in different care settings [41]. The algorithm automatically selects covariates based on their confounding potential and has been shown to improve adjustment for unmeasured confounding [41]. Fourth, we conducted bias analyses in which we re-estimated treatment effects after adjusting for HbA1c or eGFR to check if our estimates were robust even under assumptions of extreme imbalance in these unmeasured confounders between treatment groups [42].

To account for potential informative censoring, we conducted further analyses: (i) intent-to-treat (ITT) analyses which do not censor for treatment discontinuation or switching allowing maximum follow-up of up to two years, and (ii) censoring-weighted analyses which create pseudo-populations in which treatment discontinuation/switching was independent of baseline covariates [39]. Other sensitivity analyses included addressing potential exposure misclassification by varying the exposure assessment window from 60 to 30 days before censoring for treatment discontinuation, and restricting analyses to patients with at least 1 and 2 years of follow-up to account for longer follow-up time necessary for the development of cardiorenal outcomes. In these analyses, follow-up started at 1- and 2-years post-index until the end of available follow-up. We also compared empagliflozin with each individual GLP-1RA agent (liraglutide or dulaglutide) after re-matching them and re-estimating the PS for each pair.

All analyses were performed using the Aetion Evidence Platform® (2023), a software for real-world data analysis validated for a range of studies (Aetion, Inc.) [43], with R version 4.2 (R Foundation for Statistical Analysis) and SAS 9.4 Statistical Software (SAS Institute Inc., Cary, NC).

## Results

The study population included 169,599 patients with T2D initiating empagliflozin and 298,298 initiating GLP-1RA prior to matching. After 1:1 matching, 141,541 patients remained in each group (Additional file 1: sFigure 2).

Mean ages of empagliflozin and GLP-1RA initiators were similar even prior to matching: 63 vs. 62 years. Empagliflozin initiators were less likely to be female (43% vs. 54%) and white (71% vs. 75%). The proportion of patients with baseline CVD was approximately similar (35% vs. 33%). Empagliflozin initiators were slightly more likely to have a history of metformin use at baseline (82% vs. 74%) and less likely to have a history of insulin use (21% vs. 38%) relative to GLP-1RA initiators. Empagliflozin initiators were also less likely to have a history of diabetes complications and CKD. All these differences were removed after PS matching (Table 1). Laboratory results, available in a subset of the populations, were also balanced even prior to PS matching (Table 1 and Additional file 1: sTable 3). Laboratory results were still balanced after restricting analyses to patients with non-missing HbA1c and eGFR (Additional file 1: sTable 4). Approximately 52% of the matched population were older adults ≥ 65 years (Table 1). The c-statistic of the model predicting treatment as a function of covariates in the post-matched database was ~ 0.5 indicating satisfactory balance.

**Table 1:** Selected baseline characteristics of patients with type 2 diabetes initiating empagliflozin or GLP-1RA

	Before matching			After matching		
	Empagliflozin *N* = 169,599 (%)	GLP-1RA *N* = 298,298 (%)	St. Diff	Empagliflozin *N* = 141,541 (%)	GLP-1RA *N* = 141,541 (%)	St. Diff
*Demographics*						
Age, years [mean (SD)]	63.10 (8.62)	62.34 (8.71)	-0.0877	62.57 (8.64)	62.62 (8.65)	0.0058
Sex (Female)	73,320 (43.2%)	162,186 (54.4%)	0.2255	65,824 (46.5%)	65,369 (46.2%)	-0.0060
Race categories*						
…White	78,748 (70.6%)	145,670 (75.1%)	0.1013	66,543 (72.9%)	66,387 (72.7%)	-0.0045
…Black	10,932 (9.8%)	20,744 (10.7%)	0.0297	9,330 (10.2%)	9,313 (10.2%)	0.0000
…Asian	5,637 (5.1%)	4,872 (2.5%)	-0.1363	3,176 (3.5%)	3,253 (3.6%)	0.0054
…Hispanic	10,501 (9.4%)	14,957 (7.7%)	-0.0608	8,152 (8.9%)	8,141 (8.9%)	0.0000
…Other or unknown	5,663 (5.1%)	7,814 (4.0%)	-0.0528	4,134 (4.5%)	4,241 (4.6%)	0.0048
*Burden of comorbidities*						
Combined comorbidity score^†^ [mean (SD)]	1.16 (1.66)	1.40 (1.76)	0.1403	1.17 (1.66)	1.19 (1.63)	0.0122
Frailty Score [mean (SD)]	0.16 (0.04)	0.17 (0.04)	0.2500	0.16 (0.04)	0.16 (0.04)	0.0000
*Lifestyle-related factors*						
Obesity	62,936 (37.1%)	142,318 (47.7%)	0.2157	57,224 (40.4%)	57,072 (40.3%)	-0.0020
Smoking	30,693 (18.1%)	57,606 (19.3%)	0.0308	25,682 (18.1%)	25,745 (18.2%)	0.0026
Alcohol abuse or dependence	1,908 (1.1%)	2,993 (1.0%)	-0.0098	1,492 (1.1%)	1,558 (1.1%)	0.0000
*Diabetes complications*						
Diabetic nephropathy	21,758 (12.8%)	52,661 (17.7%)	0.1366	19,041 (13.5%)	19,333 (13.7%)	0.0058
Diabetic retinopathy	17,559 (10.4%)	36,210 (12.1%)	0.0538	14,902 (10.5%)	14,956 (10.6%)	0.0033
Diabetic neuropathy	35,709 (21.1%)	76,604 (25.7%)	0.1088	31,042 (21.9%)	31,485 (22.2%)	0.0072
Hypoglycemia	18,263 (10.8%)	33,505 (11.2%)	0.0128	15,028 (10.6%)	15,186 (10.7%)	0.0032
Hyperglycemia	83,573 (49.3%)	149,606 (50.2%)	0.0180	70,774 (50.0%)	71,025 (50.2%)	0.0040
Diabetic ketoacidosis	541 (0.3%)	1,387 (0.5%)	0.0317	478 (0.3%)	494 (0.3%)	0.0000
Hyperosmolar hyperglycemic nonketotic syndrome	1,473 (0.9%)	2,893 (1.0%)	0.0103	1,246 (0.9%)	1,240 (0.9%)	0.0000
*Diabetes medications*						
No. of glucose-lowering medications on cohort entry^‡^ [mean (SD)]	1.47 (0.94)	1.38 (0.95)	-0.0952	1.43 (0.94)	1.44 (0.95)	0.0106
Metformin	139,424 (82.2%)	220,334 (73.9%)	-0.2015	114,420 (80.8%)	114,619 (81.0%)	0.0051
Sulfonylureas − 2nd generation	67,731 (39.9%)	116,640 (39.1%)	-0.0164	57,505 (40.6%)	57,294 (40.5%)	-0.0020
Thiazolidinediones (TZD)	15,423 (9.1%)	26,796 (9.0%)	-0.0035	13,086 (9.2%)	13,101 (9.3%)	0.0035
DPP-4i	62,785 (37.0%)	82,214 (27.6%)	-0.2020	47,299 (33.4%)	47,962 (33.9%)	0.0106
Insulins	34,945 (20.6%)	113,210 (38.0%)	0.3895	33,966 (24.0%)	34,650 (24.5%)	0.0117
*Comorbidities*						
Baseline CVD	58,858 (34.7%)	98,937 (33.2%)	-0.0317	46,906 (33.1%)	46,904 (33.1%)	0.0000
Acute MI	4,118 (2.4%)	5,315 (1.8%)	-0.0419	2,901 (2.0%)	2,901 (2.0%)	0.0000
Unstable angina	5,173 (3.1%)	7,080 (2.4%)	-0.0428	3,713 (2.6%)	3,715 (2.6%)	0.0000
Coronary procedure	4,663 (2.7%)	5,050 (1.7%)	-0.0682	3,014 (2.1%)	3,029 (2.1%)	0.0000
Heart failure	14,415 (8.5%)	28,221 (9.5%)	0.0349	11,649 (8.2%)	11,771 (8.3%)	0.0036
Ischemic stroke	13,366 (7.9%)	22,506 (7.5%)	-0.0150	10,683 (7.5%)	10,653 (7.5%)	0.0000
Peripheral arterial disease	14,605 (8.6%)	27,478 (9.2%)	0.0211	12,117 (8.6%)	12,072 (8.5%)	-0.0036
Acute Kidney Injury	4,426 (2.6%)	13,057 (4.4%)	0.0981	3,975 (2.8%)	4,067 (2.9%)	0.0060
CKD Stages 1–2	6,139 (3.6%)	12,264 (4.1%)	0.0260	5,087 (3.6%)	5,173 (3.7%)	0.0053
CKD Stages 3–4	11,652 (6.9%)	40,195 (13.5%)	0.2194	11,001 (7.8%)	11,261 (8.0%)	0.0074
CKD unspecified	4,874 (2.9%)	17,781 (6.0%)	0.1508	4,985 (3.5%)	5,132 (3.6%)	0.0054
Electrolyte disorders	9,714 (5.7%)	21,438 (7.2%)	0.0611	8,242 (5.8%)	8,350 (5.9%)	0.0043
Disorders of fluid balance	4,718 (2.8%)	10,430 (3.5%)	0.0401	3,985 (2.8%)	3,989 (2.8%)	0.0000
COPD	13,986 (8.2%)	28,471 (9.5%)	0.0458	11,842 (8.4%)	11,958 (8.4%)	0.0000
Obstructive sleep apnea	29,185 (17.2%)	66,566 (22.3%)	0.1284	26,246 (18.5%)	26,285 (18.6%)	0.0026
Fractures	2,521 (1.5%)	5,352 (1.8%)	0.0236	2,199 (1.6%)	2,213 (1.6%)	0.0000
NASH/NAFLD	10,549 (6.2%)	19,469 (6.5%)	0.0123	9,095 (6.4%)	9,161 (6.5%)	0.0041
Liver disease	5,979 (3.5%)	10,293 (3.5%)	0.0000	4,871 (3.4%)	4,865 (3.4%)	0.0000
Dementia	4,114 (2.4%)	8,159 (2.7%)	0.0190	3,460 (2.4%)	3,469 (2.5%)	0.0065
*Other Medications*						
ACEI and ARBs	126,421 (74.5%)	222,629 (74.6%)	0.0023	105,435 (74.5%)	105,697 (74.7%)	0.0046
Beta-blockers	64,467 (38.0%)	114,431 (38.4%)	0.0082	52,505 (37.1%)	52,849 (37.3%)	0.0041
Thiazide and thiazide-like diuretics	22,002 (13.0%)	45,473 (15.2%)	0.0632	19,162 (13.5%)	19,134 (13.5%)	0.0000
Loop diuretics	19,517 (11.5%)	49,735 (16.7%)	0.1498	17,285 (12.2%)	17,625 (12.5%)	0.0091
Mineralocorticoid receptor antagonists	6,729 (4.0%)	14,863 (5.0%)	0.0483	5,750 (4.1%)	5,743 (4.1%)	0.0000
Antiarrhythmics	2,819 (1.7%)	4,989 (1.7%)	0.0000	2,277 (1.6%)	2,317 (1.6%)	0.0000
Anticoagulants	11,921 (7.0%)	21,884 (7.3%)	0.0116	9,674 (6.8%)	9,734 (6.9%)	0.0040
PCSK9 inhibitors and other lipid-lowering agents	27,069 (16.0%)	46,642 (15.6%)	-0.0110	21,962 (15.5%)	22,251 (15.7%)	0.0055
Corticosteroids	29,454 (17.4%)	57,153 (19.2%)	0.0466	25,378 (17.9%)	25,507 (18.0%)	0.0026
Opioids	49,528 (29.2%)	105,449 (35.4%)	0.1329	43,272 (30.6%)	43,662 (30.8%)	0.0043
*Healthcare utilization*						
Internist (-30 days to cohort entry)	110,466 (65.1%)	187,744 (62.9%)	-0.0458	91,622 (64.7%)	91,748 (64.8%)	0.0021
Endocrinologist (-30 days to cohort entry)	23,709 (14.0%)	54,114 (18.1%)	0.1119	20,990 (14.8%)	21,223 (15.0%)	0.0056
Cardiologist (-30 days to cohort entry)	19,830 (11.7%)	26,889 (9.0%)	-0.0887	14,264 (10.1%)	14,379 (10.2%)	0.0033
No. of hospitalizations [mean (SD)]	0.14 (0.46)	0.15 (0.48)	0.0213	0.13 (0.45)	0.13 (0.45)	0.0000
LOS (-30 days to cohort entry) [mean (SD)]	0.09 (0.89)	0.07 (0.76)	-0.0242	0.08 (0.82)	0.09 (0.78)	0.0125
No. of emergency visits [mean (SD)]	0.43 (1.23)	0.50 (1.31)	0.0551	0.44 (1.24)	0.45 (1.26)	0.0080
No. of distinct brand medications [mean (SD)]	2.80 (1.70)	3.21 (1.93)	0.2254	2.85 (1.72)	2.88 (1.76)	0.0172
Out-of-pocket pharmacy cost ($)^§^ [mean (SD)]	730.06 (780.06)	814.76 (866.16)	0.1028	740.42 (790.32)	752.85 (789.26)	0.0157
*Laboratory results* ^||^						
HbA1c, mean (SD)	9.0(2.31)	9.0(2.41)	0.016	9.0(2.31)	9.0(2.39)	0.001
Creatinine, mean (SD)	1.0(0.47)	1.0(0.50)	0.077	1.0(0.47)	1.0(0.47)	0.059
eGFR, mean (SD)	84.4(21.95)	81.6(24.92)	0.119	84.5(22.31)	83.0(23.33)	0.066
UACR, mean (SD)	107.8(520.79)	154.2(610.36)	0.082	112.1(548.33)	126.7(536.22)	0.027
LDL, mean (SD)	98.7(44.15)	99.9(43.34)	0.029	99.5(44.38)	98.5(43.16)	0.023
HDL, mean (SD)	46.9(16.87)	47.0(17.09)	0.007	46.8(16.68)	46.5(16.73)	0.018
Triglyceride, mean (SD)	206.2(163.88)	206.1(156.88)	0.001	208.2(165.01)	206.8(158.86)	0.009

ACEI: angiotensin converting enzyme inhibitors; ARB: angiotensin receptor blockers; CKD: chronic kidney disease; COPD: chronic obstructive pulmonary disease; DM: diabetes mellitus; EMPA: empagliflozin; GLP-1RA: glucagon like peptide-1 receptor agonists; HbA1c: hemoglobin A1c; MI: myocardial infarction; PCSK9: proprotein convertase subtilisin/kexin type 9 serine protease; PS: propensity score; SD: standard deviation; St. Diff: absolute standardized mean differences (< 0.1 was suggested as a measure of satisfactory balance as in Austin PC. Assessing covariate balance when using the generalized propensity score with quantitative or continuous exposures. Stat Methods Med Res. 2019 May;28(5):1365–1377. doi: 10.1177/0962280218756159. Epub 2018 Feb 8. PMID: 29,415,624; PMCID: PMC6484705.)

Baseline characteristics were measured during 12 months prior to and including the index date (cohort entry date) unless otherwise stated

* Race information is only available in Medicare and Clinformatics® and not reported in Marketscan

† Calculated using the weights in Gagne JJ, Glynn RJ, Avorn J, Levin R, Schneeweiss S. A combined comorbidity score predicted mortality in elderly patients better than existing scores. J Clin Epidemiol. 2011 Jul;64(7):749 − 59. doi: 10.1016/j.jclinepi.2010.10.004. Epub 2011 Jan 5. PMID: 21,208,778; PMCID: PMC3100405

‡ Number of diabetes medications calculated here did not include the index medications

§ Includes deductibles, copayments, and coinsurance

|| Laboratory results were available in Clinformatics® (approximately 45%) and MarketScan (approximately 5–10%) databases, thus not included in the PS model

The median follow-up time after matching was 5 months (interquartile range: 3–10 months) for both empagliflozin and GLP-1RA initiators. Approximately 21–22% of the original PS-matched cohort had follow-up of at least one year and 6–7% remained at two years. The most common reason for censoring was treatment discontinuation (40–45%) for both treatment groups (Additional file 1: sTable 5).

### Cardiovascular effectiveness outcomes

After matching, rates of the composite of MI or stroke were similar between empagliflozin and GLP-1RA initiators [13.1 and 13.4 events per 1,000 person-years (PY) with corresponding HR of 0.99 (0.92, 1.07) and RD of -0.23 (-1.25, 0.79) per 1,000 PY]. Empagliflozin was associated with lower rates of HHF relative to GLP-1RA with rates of 5.0 and 7.3 per 1,000 PY respectively, corresponding to HR of 0.69 (0.62, 0.77) and RD of -2.28 (-2.98, -1.59).

In analyses restricted to Medicare, empagliflozin was associated with a slightly lower risk of MACE compared with GLP-1RA initiators, based on rates of 22.6 and 25.1 per 1,000 PY respectively, HR of 0.90 (0.82, 0.99), and RD of -2.54 (-4.76, -0.32) per 1,000 PY. Rates of the composite of cardiovascular mortality or HHF were 14.2 and 18.3 events per 1,000 PY among empagliflozin and GLP-1RA initiators, respectively [HR: 0.77 (0.69, 0.86), RD: -4.11 (-5.95, -2.29) per 1,000 PY]. In a subgroup of patients with history of baseline CKD stages 3–4 (10,837 PS-matched pairs), empagliflozin was associated with a lower risk of ESKD compared with GLP-1RA initiators [HR: 0.75 (0.60, 0.94), RD: -6.77 (-11.97, -1.61) per 1,000 PY] (Table 2).

**Table 2:** Comparative risk of cardiorenal outcomes among 1:1 PS-matched initiators of empagliflozin vs. GLP-1RA

	Empagliflozin	GLP-1RA	Empagliflozin vs. GLP-1RA	
Primary and secondary outcomes	N events (IR/1,000 PY)	N events (IR/1,000 PY)	HR (95% CI)	RD/1,000PY (95% CI)
Empagliflozin vs. GLP-1RA (N of matched pairs = 141,541)				
**Primary outcomes**				
Composite of myocardial infarction or stroke	1317 (13.12)	1274 (13.35)	0.99 (0.92, 1.07)	-0.23 (-1.25, 0.79)
Hospitalization for heart failure	507 (5.03)	700 (7.31)	0.69 (0.62, 0.77)	-2.28 (-2.98, -1.59)
MACE*^†^	854 (22.6)	922 (25.14)	0.90 (0.82, 0.99)	-2.54 (-4.76, -0.32)
Composite of cardiovascular mortality or hospitalization for heart failure^†^	540 (14.22)	675 (18.34)	0.77 (0.69, 0.86)	-4.11 (-5.95, -2.29)
**Secondary outcomes**				
Composite of primary outcomes (all databases)^‡^	1757 (17.54)	1881 (19.77)	0.89 (0.84, 0.95)	-2.23 (-3.45, -1.02)
Composite of primary outcomes (Medicare)^§^	1158 (30.75)	1305 (35.76)	0.86 (0.79, 0.93)	-5.01 (-7.65, -2.39)
Hospitalization for heart failure (broad)^||^	2301 (23.02)	2600 (27.44)	0.85 (0.80, 0.90)	-4.42 (-5.83, -3.01)
Myocardial infarction	813 (8.08)	815 (8.52)	0.95 (0.86, 1.05)	-0.44 (-1.25, 0.36)
Stroke	522 (5.18)	466 (4.86)	1.08 (0.95, 1.22)	0.32 (-0.31, 0.94)
Cardiovascular mortality^†^	202 (5.3)	242 (6.54)	0.81 (0.67, 0.97)	-1.24 (-2.34, -0.14)
All-cause mortality ^†^	517 (13.57)	570 (15.4)	0.88 (0.78, 0.99)	-1.83 (-3.56, -0.11)
Unstable angina	263 (2.61)	259 (2.7)	0.96 (0.81, 1.14)	-0.09 (-0.55, 0.36)
Coronary revascularization	990 (9.86)	958 (10.03)	0.99 (0.90, 1.08)	-0.17 (-1.06, 0.71)
ESKD^#^	128 (19.73)	180 (26.5)	0.75 (0.60, 0.94)	-6.77 (-11.97, -1.61)

ESKD: end-stage kidney disease; PS: propensity score; IR: Incidence rate; PY: person-years; HR: hazard ratio; CI: confidence intervals; RD: rate difference

* MACE includes hospitalizations for myocardial infarction, stroke, or cardiovascular mortality

^†^ Only available in the Medicare database (54,292 1:1 PS-matched pairs)

^‡^ Includes hospitalizations for myocardial infarction, stroke, or heart failure

^§^ Includes hospitalizations for myocardial infarction, stroke, cardiovascular mortality, or heart failure

|| Includes hospitalization for heart failure in all discharge positions

# Restricted to patients with chronic kidney disease stages 3–4 (10,837 1:1 PS-matched pairs)

Estimates for secondary outcomes were overall consistent, with similar risks of MI, stroke, unstable angina, and coronary revascularization between the groups, while the risks of composite of primary outcomes, HHF (defined more broadly), all-cause, and cardiovascular mortality were lower in empagliflozin vs. GLP-1RA initiators (Table 2). Empagliflozin was also associated with lower risks of the composite of MI, stroke, or HHF in all patients, and the composite of MACE or HHF in older Medicare patients relative to GLP-1RA.

Database-specific estimates were overall consistent except for small differences in commercial claims databases due to the small numbers of events (Additional file 1: sTable 6).

**Fig. 1:**
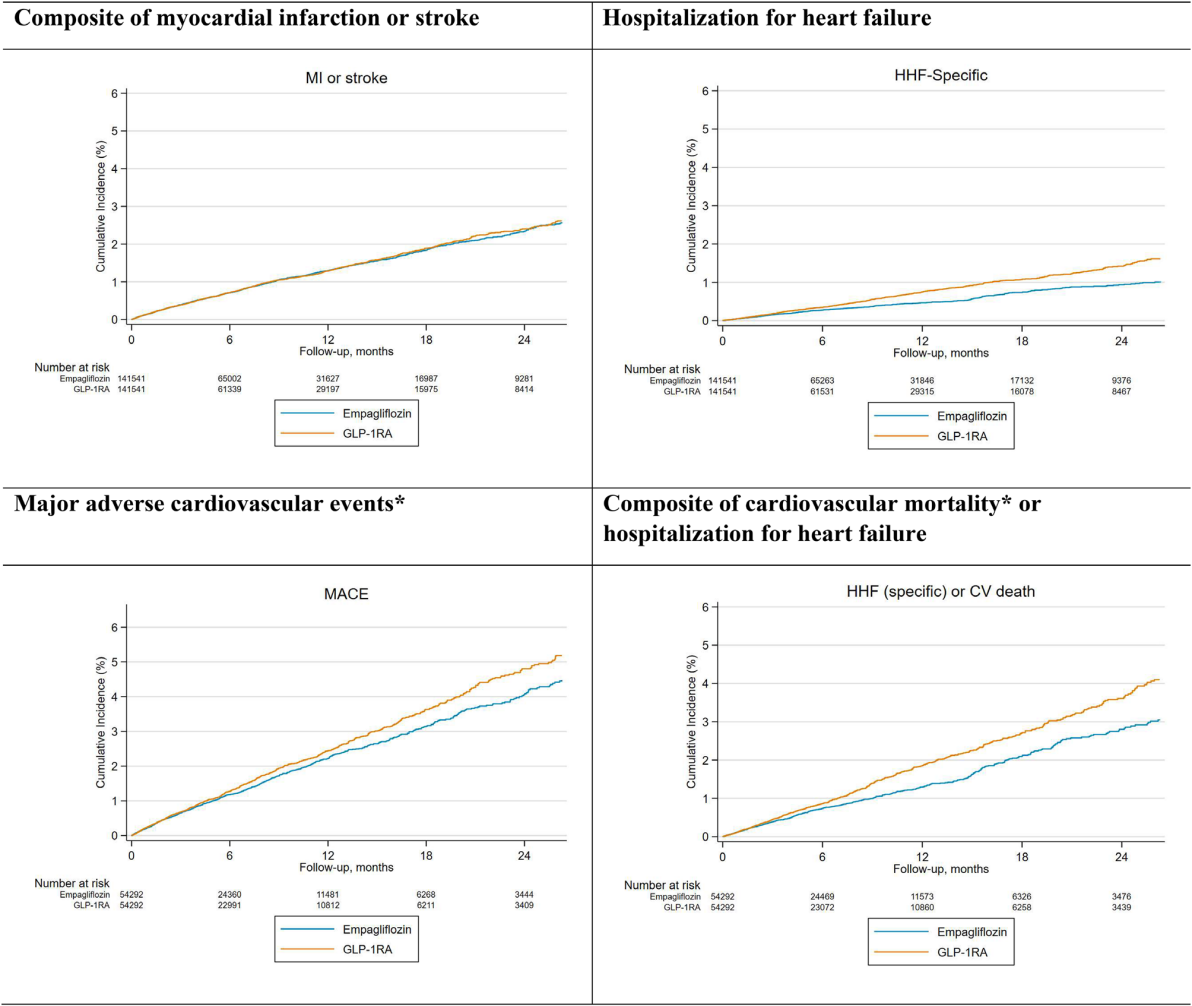
Cumulative risk of primary outcomes among PS-matched initiators of empagliflozin vs. GLP-1RA CAPTION: The risks of cardiovascular mortality, MACE, and HHF were lower in the empagliflozin vs. GLP-1RA initiators. Cardiovascular mortality data was only available in the Medicare database CV: cardiovascular; GLP-1RA: Glucagon-like peptide-1 receptor agonists; MACE: major adverse cardiovascular events; PS: propensity score

Consistent with HR and RD estimates, CIF curves showed similar risks of the composite of MI or stroke, and lower risk of MACE among patients initiating empagliflozin relative to GLP-1RA. The risks of HHF and the composite outcome of cardiovascular mortality or HHF were also lower in empagliflozin relative to GLP-1RA initiators. (Fig. 1).

### Subgroup analyses

**Fig. 2:**
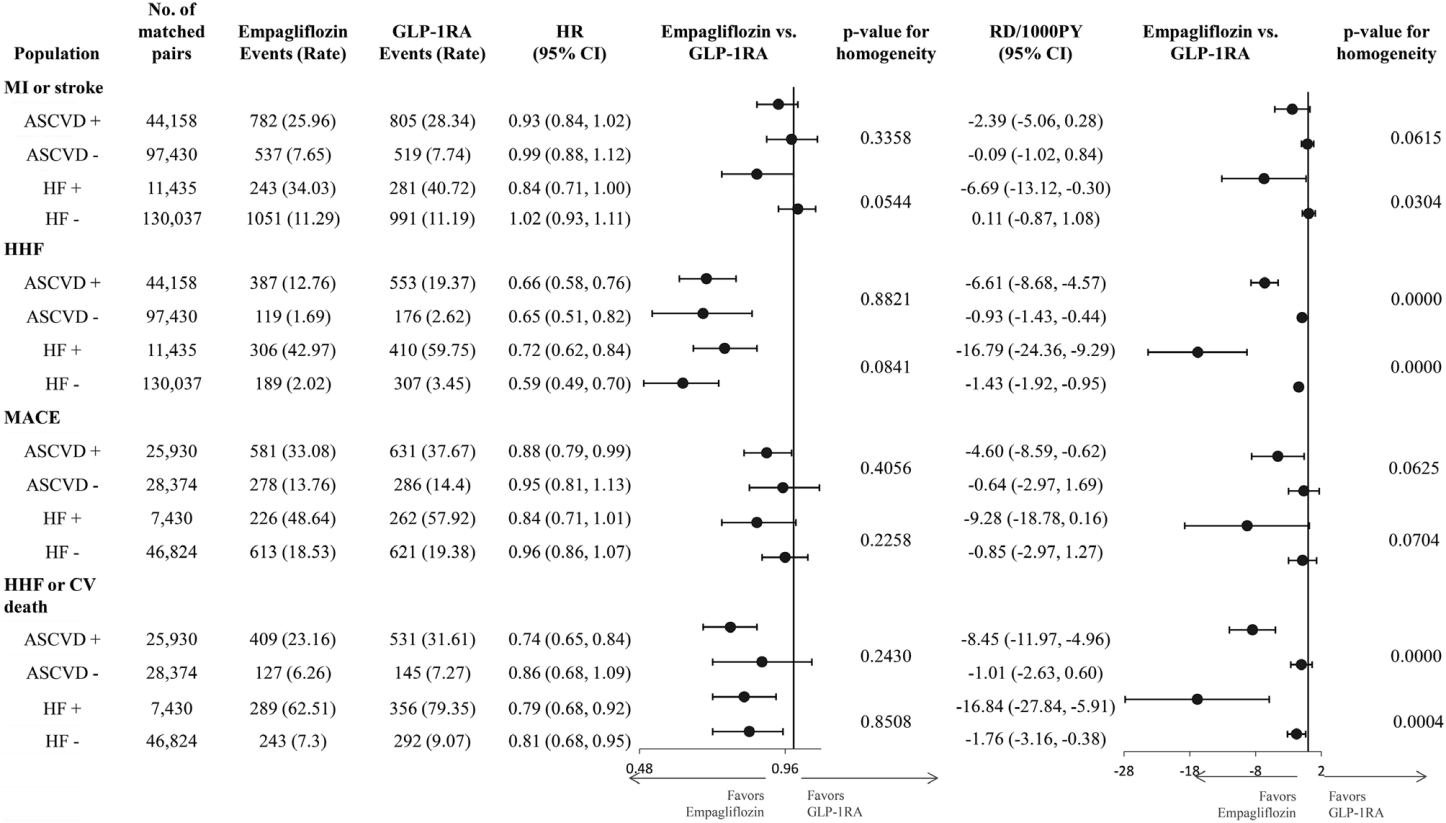
Subgroup analyses for primary outcomes by atherosclerotic cardiovascular disease or heart failure CAPTION: On the relative scale, HRs were consistent across all subgroups examined for all outcomes. On the absolute scale, for all outcomes, RDs were larger in patients with ASCVD compared to those without it, and in those with HF compared to those without it

On the relative scales, estimates for the composite outcome of MI or stroke and MACE outcomes were similar in patients with and without baseline history of ASCVD or HF. Relative risk reductions in HHF and the composite of HHF and cardiovascular mortality were consistently observed independently of baseline ASCVD and HF. For all outcomes, absolute RDs were larger in patients with baseline ASCVD or HF than in those without these conditions (Fig. 2).

**Fig. 3:**
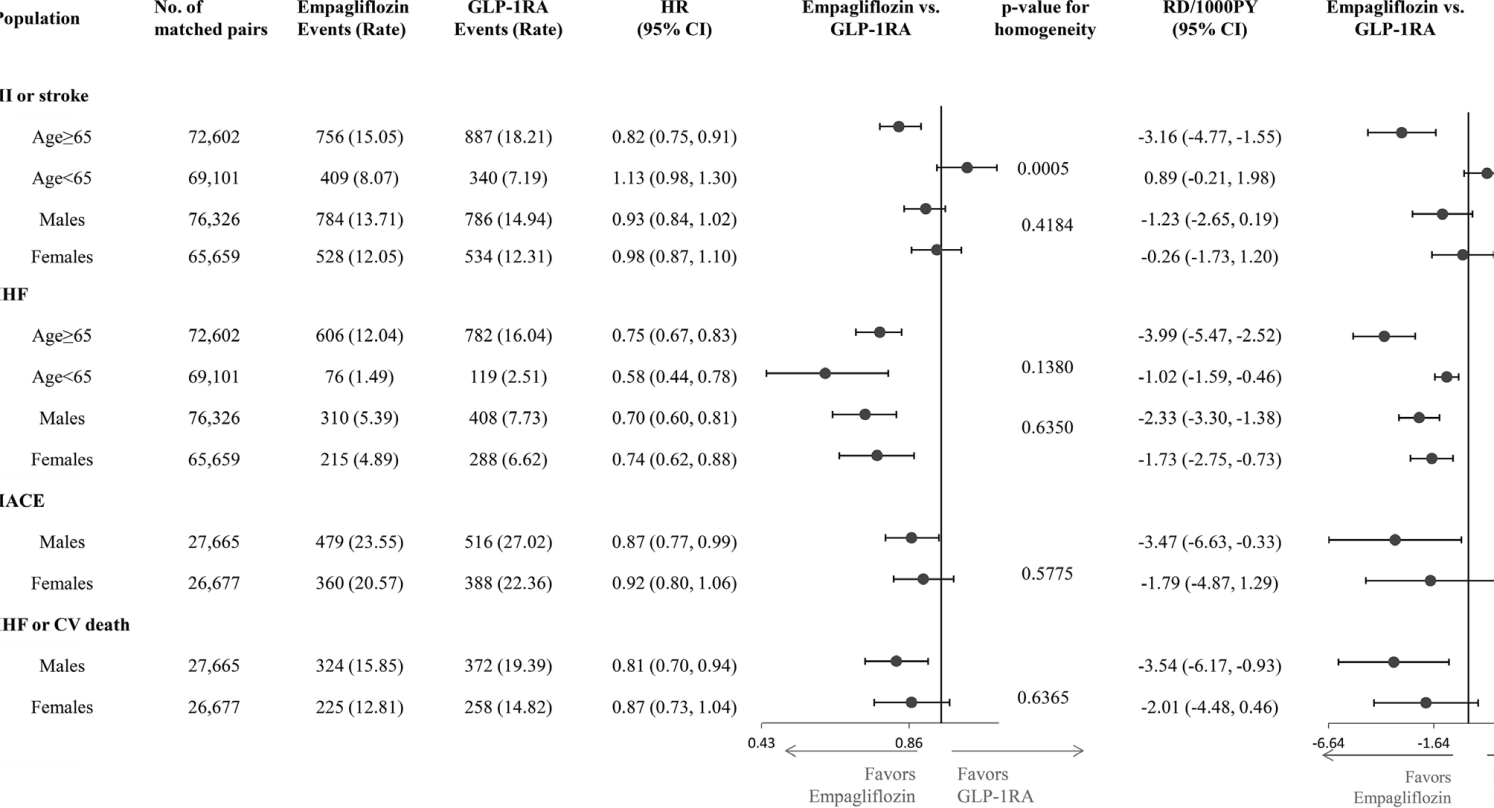
Subgroup analyses for primary outcomes by age and sex CAPTION: On the relative scale, empagliflozin was associated with a lower risk of MI/stroke in patients 65 years or older, while it was not associated with MI/stroke in patients younger than 65 years. The HR estimates were consistent across other subgroups for all outcomes. For all outcomes, RD estimates were larger in older than in younger patients, while they did not differ by sex

Stratified analyses by age showed that the relative risk of the composite outcome of MI or stroke was lower in older vs. younger patients, while the relative risk reductions for HHF were similar across age categories. Estimates for the remaining outcomes did not differ by age subgroups. Stratified analyses by sex produced similar relative hazards between male and female patients across all outcomes. Absolute RDs were larger in older vs. younger patients (Fig. 3). Subgroup analyses for secondary outcomes provided similar findings (Additional file 1: sTable 7).

### Sensitivity analyses

Sensitivity analyses were overall consistent with primary analytical findings (Additional file 1: sTable 8). Rematching populations using laboratory results, high-dimensional PS matching (Additional file 1: sTables 9–10), and bias analyses (Additional file 1: sFigures 3, 4) supported the robustness of the primary findings to unmeasured confounding. Analyses restricted to patients with ≥ 1–2 years of follow-up and comparing empagliflozin vs. each individual GLP-1RA agent (liraglutide or dulaglutide) revealed similar findings (Additional file 1: sTables 11, 12).

## Discussion

In this comparative effectiveness cohort study, empagliflozin was associated with a similar risk of a composite of MI or stroke, and a lower risk of HHF, MACE, and a composite of HHF or cardiovascular mortality, when compared with cardioprotective GLP-1RA agents (i.e., liraglutide, albiglutide, dulaglutide, or semaglutide). In a subgroup of patients with T2D and baseline CKD stages 3–4, empagliflozin was associated with a lower risk of ESKD relative to GLP-1RA. Regarding the secondary outcomes, patients initiating empagliflozin had a lower risk of all-cause and cardiovascular mortality, compared to cardioprotective GLP-1RA, whereas the risks of MI and stroke (individually considered) were comparable between exposure groups. These analyses remained robust to unmeasured confounding due to eGFR and HbA1c in analyses restricted to patients with available laboratory results and in bias analyses quantifying the potential impact of unmeasured confounding. Across the pre-specified subgroups, similar results were found, but absolute benefits of empagliflozin were larger in patients with history of ASCVD or HF and in older adults.

Overall, our findings were in line with previous studies comparing empagliflozin and GLP-1RA agents [19, 20], although our study focused on GLP-1RA agents that demonstrated cardioprotective effects in trials (without considering exenatide and lixisenatide). Unlike previous studies, we evaluated the risk of cardiovascular mortality, an outcome that remains relatively unexplored in clinical practice and an approved indication for empagliflozin.

The relative effect of empagliflozin and GLP-1RA on MI and stroke outcomes is an area of debate in the literature. GLP-1RA agents, with the exception of exenatide and lixisenatide, offered risk reductions relative to placebo for MI and stroke by ~ 10–17% in patients with T2D, with estimates varying across different trials [2]. Empagliflozin, on the other hand, showed a 13% risk reduction for MI relative to placebo which did not reach statistical significance, and a numerical increase in the risk of stroke in patients with T2D and ASCVD [1]. Observational studies comparing empagliflozin with GLP-1RA also reported HRs ranging from 0.9 to 1.0 with larger risk reductions in patients with history of cardiovascular events [19, 20]. In our study, we found no association between empagliflozin vs. cardioprotective GLP-1RA and the risk of MI or stroke outcomes (either as a composite outcome or individually considered), in line with the placebo-controlled trials and prior evidence [2, 3, 19, 20]. We observed that the absolute risk reductions of empagliflozin for MI and stroke were larger in patients with history of HF and in patients 65 years or older, highlighting the fact that these patient subgroups could potentially have greater benefit from empagliflozin relative to cardioprotective GLP-1RA agents [40].

While the benefits of empagliflozin on HHF outcomes have been well-demonstrated, the effect of GLP-1RA on HHF from placebo-controlled trials was modest, at 11% relative risk reduction, and not uniformly established across different trials [1, 2]. We found a 31% relative risk reduction of empagliflozin relative to GLP-1RA, which was consistent across multiple pre-defined subgroups of patients and in line with prior evidence [19, 20]. Absolute risk reductions were larger in patients with history of ASCVD or HF and in older adults, again highlighting the role of empagliflozin in reducing the risk of HHF in these patients [40].

In our study, empagliflozin was associated with a 10% relative risk reduction for MACE compared to GLP-1RA. Absolute risk reductions were larger in patients with history of ASCVD or HF than in patients without it. This is consistent with current evidence from placebo-controlled trials and prior observational studies [19, 20]. This small benefit of empagliflozin towards MACE was mainly driven by the 19% reduction in the risk of cardiovascular mortality. While we have no trial evidence on the relative benefits of empagliflozin vs. GLP-1RA initiators with respect to cardiovascular mortality, in placebo-controlled trials empagliflozin offered apparently larger relative risk reductions than GLP-1RA versus placebo (38% vs. 22% risk reductions respectively), mainly in patients with T2D and history of ASCVD [1, 2]. The absolute risk reductions in MACE observed in the present study were larger in patients with history of ASCVD or HF, suggesting that patients in these high-risk subgroups could benefit more from empagliflozin relative to cardioprotective GLP-1RA agents.

Our study is one of the few to compare the renal benefits of empagliflozin and GLP-1RA. In placebo-controlled trials, empagliflozin demonstrated a 46% relative risk reduction for a composite kidney outcome (nephropathy including macroalbuminuria and ESKD) in patients with T2D and ASCVD, and a 28% risk reduction for kidney disease progression (ESKD) or cardiovascular mortality in patients with established CKD, consistently across subgroups defined by baseline eGFR [1, 3, 44]. GLP-1RA also demonstrated benefits on a composite nephropathy outcome [HR: 0.79 (0.73, 0.87)] in patients with T2D, with HR estimates ranging from 0.64 to 0.85 with varying degrees of precision across trials [2]. Consistent with evidence from trials suggesting strong kidney benefit of SGLT2i and weaker benefit of GLP-1 RA, in an analysis restricted to patients with history of CKD stages 3–4, we observed a 25% risk reduction of empagliflozin towards ESKD outcomes relative to GLP-1RA [1–3, 40]. However, kidney outcome trials of GLP-1 RA agents (e.g., injection semaglutide) are ongoing (FLOW trial) [45].

### Limitations

Several limitations should be considered. First, we cannot exclude unmeasured confounding. After PS matching using laboratory results in a subset of the cohort with laboratory results available, findings remained consistent with the primary analyses. Our 1:1 PS matched design incorporated a rich set of claims-based variables that have been shown to balance laboratory results and clinical parameters typically only available in electronic health records [46]. Second, our outcome definitions relied on claims-based algorithms previously validated to have high specificity and PPV but low sensitivity [39]. Third, the median follow-up was short due to the lower persistence on treatments of patients in routine clinical practice compared to RCTs. Fourth, the primary analysis may suffer from informative censoring, which we addressed using ITT and censoring-weighted analyses. Finally, beneficial effects of GLP-1RA may require longer follow-up time to become apparent especially the effects mediated by atherosclerosis progression and weight loss. Analyses restricted to patients with ≥ 1–2 years of available follow-up provided consistent findings.

## Conclusion

In this final-year report from the EMPRISE study, after extensive confounding control, empagliflozin was associated with similar risks of MI/stroke and lower risks of MACE, HHF, a composite of cardiovascular mortality or HHF, all-cause of mortality, and ESKD (in patients with CKD), when compared with selected GLP-1RA agents that demonstrated cardioprotective effects. Cardiovascular benefits of empagliflozin were larger in older patients and in patients with ASCVD or HF on the absolute scale. Our findings complement existing trial evidence by directly comparing empagliflozin with alternative cardioprotective agents and incorporating broad patient populations in clinical practice using robust and generalizable methodology.

### Electronic supplementary material

Below is the link to the electronic supplementary material.

Supplementary Material 1

## Data Availability

Due to the data user agreements with the Centers for Medicare and Medicaid Services, Optum, and Marketscan, we cannot share the analytical datasets. Databases used in the study are available to purchase from the data vendors.

## Abbreviations

ASCVD: Atherosclerotic cardiovascular disease
ASD: Absolute standardized mean differences
CKD: Chronic kidney disease
CVD: Cardiovascular disease
ESKD: End-stage kidney disease
GLP-1RA: Glucagon-like peptide-1 receptor agonists
HHF: Hospitalization for heart failure
HR: Hazard ratios
MACE: Major adverse cardiovascular events
MI: Myocardial infarction
PS: Propensity scores
SGLT-2i: Sodium-glucose cotransporter 2 inhibitors
T2D: Type 2 diabetes
RD: Rate differences
