# Correction: Cardiorenal effectiveness of empagliflozin vs. glucagon-like peptide-1 receptor agonists: final-year results from the EMPRISE study

**DOI:** 10.1186/s12933-024-02190-6

**Published:** 2024-03-18

**Authors:** Phyo T. Htoo, Helen Tesfaye, Sebastian Schneeweiss, Deborah J. Wexler, Brendan M. Everett, Robert J. Glynn, Niklas Schmedt, Lisette Koeneman, Anouk Déruaz-Luyet, Julie M. Paik, Elisabetta Patorno

**Affiliations:** 1 Division of Pharmacoepidemiology and Pharmacoeconomics, Department of Medicine, Brigham and Women’s Hospital and Harvard Medical School, 1620 Tremont Street, Suite 3030, Boston, MA 02120 USA; 2 Massachusetts General Hospital Diabetes Center, Harvard Medical School, Boston, USA; 3 Divisions of Cardiovascular and Preventive Medicine, Department of Medicine, Brigham and Women’s Hospital, Harvard Medical School, 75 Francis Street, Boston, MA USA; 4 Global Epidemiology, Boehringer Ingelheim International GmbH (Germany) DE, Berlin, Germany; 5 Global Medical Affairs, Lilly Deutschland GmbH, Bad Homburg, Germany; 6 Division of Renal (Kidney) Medicine, Brigham and Women’s Hospital, Boston, MA USA

**Correction: Cardiovascular Diabetology (2024) 23:57** 10.1186/s12933-024-02150-0

Following publication of the original article [1], the authors noticed an error in the hazard ratio (HR) for the hospitalization for heart failure (HHF) outcome in the abstract. The numbers in the other parts of the manuscript and the tables were correct.

In abstract section, the correct sentence should read “Compared with GLP-1RA, empagliflozin was associated with similar risks of MI or stroke [HR: 0.99 (0.92, 1.07); RD: − 0.23 (− 1.25, 0.79)], and lower risks of HHF [HR: 0.69 (0.62, 0.77); RD: − 2.28 (− 2.98, − 1.59)], MACE [HR: 0.90 (0.82, 0.99); RD: − 2.54 (− 4.76, − 0.32)], cardiovascular mortality or HHF [HR: 0.77 (0.69, 0.86); RD: − 4.11 (− 5.95, − 2.29)], and ESKD [0.75 (0.60, 0.94); RD: − 6.77 (− 11.97, − 1.61)].”

Figure 3 was also cut off on the right side with some columns missing which has now been corrected (Fig. 3).

**Fig. 3:**
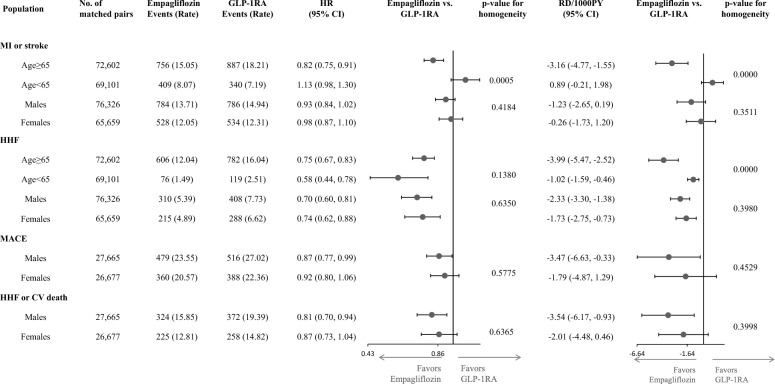
Subgroup analyses for primary outcomes by age and sex. CAPTION: On the relative scale, empagliflozin was associated with a lower risk of MI/stroke in patients 65 years or older, while it was not associated with MI/stroke in patients younger than 65 years. The HR estimates were consistent across other subgroups for all outcomes. For all outcomes, RD estimates were larger in older than in younger patients, while they did not differ by sex
