# Supplementary material for: Cardiorenal effectiveness of empagliflozin vs. glucagon-like peptide-1 receptor agonists: final-year results from the EMPRISE study
Source: Cardiovasc Diabetol. 2024 Feb 8;23:57. doi: 10.1186/s12933-024-02150-0 (PMC10854040; doi:10.1186/s12933-024-02150-0)
Supplement: Supplementary file 1 — Supplementary Material 1 [file 12933_2024_2150_MOESM1_ESM.docx]

Contents

[Supplemental Figure 1. Overview of EMPRISE study design 2](#_Toc156771625)

[Supplemental Figure 2. Flow Diagram for Empagliflozin vs GLP-1RA Cohort 3](#_Toc156771626)

[Supplemental Figure 3. Bias analysis quantifying the impact on point estimates of increasing residual associations between HbA1c and the primary cardiovascular outcomes based on observed residual differences in HbA1c between initiators of empagliflozin vs. GLP-1RA 4](#_Toc156771627)

[Supplemental Figure 4. Bias analysis quantifying the impact on point estimates of increasing residual associations between eGFR and the primary cardiovascular outcomes based on observed residual differences in eGFR between initiators of empagliflozin vs. GLP-1RA 6](#_Toc156771628)

[Supplemental Table 1. Inclusion and exclusion criteria 8](#_Toc156771629)

[Supplemental Table 2. Outcome definitions 10](#_Toc156771630)

[Supplemental Table 3. Baseline characteristics of patients initiating empagliflozin or GLP-1RA before and after 1:1 PS matching 12](#_Toc156771631)

[Supplemental Table 4. Baseline characteristics of patients initiating empagliflozin or GLP-1RA among patients with non-missing laboratory results data after 1:1 PS matching 23](#_Toc156771632)

[Supplemental Table 5. Follow-up Time and Censoring Reason for Primary Outcomes Between 1:1 PS-Matched Initiators of Empagliflozin vs GLP-1RA* 27](#_Toc156771633)

[Supplemental Table 6. Database-specific estimates for primary effectiveness outcomes between 1:1 PS-matched initiators of empagliflozin and GLP-1RA 29](#_Toc156771634)

[Supplemental Table 7. Subgroup analyses for secondary outcomes pooled across 3 databases 30](#_Toc156771635)

[Supplemental Table 8. Sensitivity analyses for 1:1 PS-matched initiators of empagliflozin vs. GLP-1RA 35](#_Toc156771636)

[Supplemental Table 9. Analyses restricted to patients with non-missing laboratory result data for the propensity score matched initiators of empagliflozin vs. GLP-1RA with respect to primary cardiovascular effectiveness outcomes* 37](#_Toc156771637)

[Supplemental Table 10. High-dimensional propensity score matched initiators of empagliflozin vs. GLP-1RA with respect to primary cardiovascular effectiveness outcomes 38](#_Toc156771638)

[Supplemental Table 11. Analyses restricted to patients with at least one and two years of follow-up data with respect to primary cardiovascular effectiveness outcomes 39](#_Toc156771639)

[Supplemental Table 12. Comparative risk of cardiorenal outcomes among 1:1 PS-matched initiators of empagliflozin vs. liraglutide or dulaglutide 41](#_Toc156771640)

# Supplemental Figure 1. Overview of EMPRISE study design


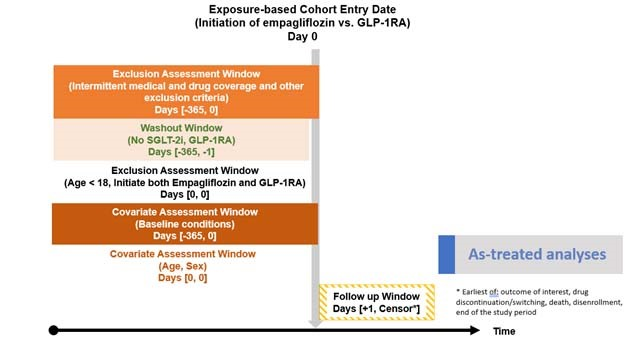


# Supplemental Figure 2. Flow Diagram for Empagliflozin vs GLP-1RA Cohort

**537,510** initiators of Empagliflozin or GLP-1RA across the 3 databases (Medicare, Clinformatics, MarketScan) between August 2014 – September 2019, having 12 months of continuous enrollment prior to cohort entry

Exclusion criteria applied during the baseline period

• 6,553 patients using exenatide or lixisenatide

• 15,059 patients using any SGLT2i

• 181 patients aged<65 years for Medicare and aged<18 years for Clinformatics and MarketScan

• 9,027 patients without a diagnosis of type 2 diabetes (T2D)

• 16,030 patients with a diagnosis of type 1 or secondary and gestational diabetes

• 18,048 patients with malignancy, ESRD, HIV, or transplant any time prior to cohort entry

• 3,209 patients with a nursing home admission

• 4 patients with >1 GLP-1RA agents on cohort entry

• 104 patients using liraglutide 3.0 mg (Saxenda)

**469,259 T2D patients** initiating Empagliflozin or GLP-1RA (170,228 Empagliflozin initiators and 299,031 GLP-1RA)

**1,362 excluded** due to loss to follow-up prior to the beginning of follow-up (i.e., 1 day after cohort entry/drug initiation date)

**298,298 T2D patients** initiating GLP-1RA

**169,599 T2D patients** initiating Empagliflozin

**141,541**

**1:1 PS-matched T2D patients** initiating Empagliflozin

**141,541**

**1:1 PS-matched T2D patients** initiating

GLP-1RA

ESRD: end-stage renal disease; GLP-1RA: glucagon-like peptide-1 receptor agonists; HIV: human immunodeficiency virus; PS: propensity score; SGLT2i: sodium-glucose cotransporter 2 inhibitors; T2D: type 2 diabetes.

# Supplemental Figure 3. Bias analysis quantifying the impact on point estimates of increasing residual associations between HbA1c and the primary cardiovascular outcomes based on observed residual differences in HbA1c between initiators of empagliflozin vs. GLP-1RA

| **Composite myocardial infarction or stroke**† | **Hospitalization for heart failure (HHF)** |
| --- | --- |
|  |  |
| **Major adverse cardiovascular events** | **Composite of cardiovascular death or HHF** |
|  |  |

HbA1c: HbA1c; GLP-1RA: dipeptidyl peptidase-4 inhibitors; RRobs: Relative risk (observed); RR_CD_: Relative risk between a 1% increase in HbA1c and the primary cardiovascular outcomes; RRadj: Relative risk fully adjusted for HbA1c.

* History of cardiovascular disease is defined as history of myocardial infarction, angina, coronary atherosclerosis and other forms of chronic ischemic heart disease, coronary procedure, heart failure, ischemic stroke, peripheral arterial disease or surgery, lower extremity amputation

† Hospitalization for myocardial infarction, or ischemic or hemorrhagic stroke

Bias analyses were based on the observed residual difference in HbA1c between initiators of empagliflozin vs. GLP-1RA (9% vs. 8%), and the observed relative risk (RR_obs_) between empagliflozin vs. GLP-1RA and the primary CVD outcomes (i.e., the primary effect estimates observed in the main analyses). The graphs plot the changes in "true" or fully adjusted relative risk (RR) for a range of associations between the observed HbA1c and the outcome (RR_CD_). RR_CD_ values were obtained from literature (Gerstein HC, Swedberg K, Carlsson J, et al. The Hemoglobin A1c Level as a Progressive Risk Factor for Cardiovascular Death, Hospitalization for Heart Failure, or Death in Patients With Chronic Heart Failure: An Analysis of the Candesartan in Heart Failure: Assessment of Reduction in Mortality and Morbidity (CHARM) Program. Arch Intern Med. 2008;168(15):1699-1704) and mean HbA1c among empagliflozin vs. comparators were also obtained from prior studies on trial-enrolled participants (Gurgle HE, White K, McAdam-Marx C. SGLT2 inhibitors or GLP-1 receptor agonists as second-line therapy in type 2 diabetes: patient selection and perspectives. Vasc Health Risk Manag. 2016;12:239-249. Published 2016 Jun 4. doi:10.2147/VHRM.S83088). Overall, fully adjusted effect estimates were fairly robust even under extreme scenarios of the association between a 1% increase in HbA1c and the primary cardiovascular outcomes.

# Supplemental Figure 4. Bias analysis quantifying the impact on point estimates of increasing residual associations between eGFR and the primary cardiovascular outcomes based on observed residual differences in eGFR between initiators of empagliflozin vs. GLP-1RA

| **Composite myocardial infarction or stroke**† | **Hospitalization for heart failure (HHF)** |
| --- | --- |
|  |  |
| **Major adverse cardiovascular events** | **Composite of cardiovascular death or HHF** |
|  |  |

HbA1c: HbA1c; GLP-1RA: dipeptidyl peptidase-4 inhibitors; RRobs: Relative risk (observed); RR_CD_: Relative risk between a 1% increase in HbA1c and the primary cardiovascular outcomes; RRadj: Relative risk fully adjusted for HbA1c.

* History of cardiovascular disease is defined as history of myocardial infarction, angina, coronary atherosclerosis and other forms of chronic ischemic heart disease, coronary procedure, heart failure, ischemic stroke, peripheral arterial disease or surgery, lower extremity amputation

† Hospitalization for myocardial infarction, or ischemic or hemorrhagic stroke

Bias analyses are based on the observed residual difference in eGFR between initiators of empagliflozin vs. GLP-1RA (85 vs. 81 mL/min/1.73m^2^), and the observed relative risk (RR_obs_) between empagliflozin vs. GLP-1RA and the primary CVD outcomes (i.e., the primary effect estimates observed in the main analyses). The graphs plot the changes in "true" or fully adjusted relative risk (RR) for a range of associations between the observed HbA1c and the outcome (RR_CD_). RR_CD_ values were obtained from literature (Sarnak MJ, Levey AS, Schoolwerth AC, et al. Kidney disease as a risk factor for development of cardiovascular disease: a statement from the American Heart Association Councils on Kidney in Cardiovascular Disease, High Blood Pressure Research, Clinical Cardiology, and Epidemiology and Prevention. Circulation. 2003;108(17):2154-2169.) and mean eGFR among empagliflozin vs. comparators were also obtained from prior studies on trial-enrolled participants (Gurgle HE, White K, McAdam-Marx C. SGLT2 inhibitors or GLP-1 receptor agonists as second-line therapy in type 2 diabetes: patient selection and perspectives. Vasc Health Risk Manag. 2016;12:239-249. Published 2016 Jun 4. doi:10.2147/VHRM.S83088). Overall, fully adjusted effect estimates were fairly robust even under extreme scenarios of the association between a 10-unit decrease in eGFR and the primary cardiovascular outcomes.

# Supplemental Table 1. Inclusion and exclusion criteria

| **Inclusion criteria** | **Codes** | **Setting/ Position** |
| --- | --- | --- |
| Type 2 diabetes mellitus | ICD 9 diagnosis: 250.00, 250.02, 250.10, 250.12, 250.20, 250.22, 250.30, 250.32, 250.40, 250.42, 250.50, 250.52, 250.60, 250.62, 250.70, 250.72, 250.80, 250.82, 250.90, 250.92  ICD 10 diagnosis: E11.x | Any setting,  any position |
| **Exclusion criteria** | **Codes** |  |
| Nursing home | Claims in SNF dataset  CPT codes: 99301, 99302, 99303, 99311, 99312, 99313, 99315, 99316, 99379, 99380, G0066  Place of service code: 31 (skilled nursing facility), 32 (nursing facility), 33 (custodial care facility) | Any setting,  any position |
| Type 1 diabetes mellitus | ICD 9 diagnosis: 250.01, 250.03, 250.11, 250.13, 250.21, 250.23, 250.31, 250.33, 250.41, 250.43, 250.51, 250.53, 250.61, 250.63, 250.71, 250.73, 250.81, 250.83, 250.91, 250.93  ICD 10 diagnosis: E10.x | Any setting,  any position |
| Secondary and gestational diabetes | ICD 9 diagnosis: 249.x, 648.8x  ICD 10 diagnosis: E08.x, E09.x, O24.4x, O99.81 | Any setting, any position |
| End-stage renal disease (including dialysis or renal transplant) | ICD-9 diagnosis: 585.5, 585.6, 996.81, V42.0, V45.1x, V56.xx  ICD-9 procedure: 39.95, 54.98, 55.6x  ICD-10 diagnosis: N18.5, N18.6, R88.0, T82.41x, T82.42x, T82.43x, T82.49x, T85.611x, T85.621x, T85.631x, T85.71x, T86.1x, Y84.1, Z48.22, Z49.xx, Z91.15, Z94.0, Z99.2  ICD-10 procedure: 0TY00Zx, 0TY10Zx, 3E1M39Z, 5A1Dx0Z  HCPCS/CPT: 50360, 50365, 90920, 90921, 90924, 90925, 90935, 90937, 90939, 90940, 90945, 90947, 90957, 90958, 90959, 90960, 90961, 90962, 90965, 90966, 90969, 90970, 90989, 90993, 90999, 90997, 99512, 99559, 99512, G0257, G0314, G0315, G0316, G0317, G0318, G0319, G0322, G0323, G0326, G0327, S9335, S9339 | Any setting, any position |
| Organ transplant | ICD-9 diagnosis: V42.1x, V42.6x, V42.7x, V42.8x (except for V42.81 or V42.82), V42.9x, V58.44, E878.0x  ICD-9 procedure: 33.5x, 33.6x, 37.51, 46.97, 50.5x, 52.8x, 55.6x, 996.8x (except for 996.85 or 996.88), V42.0x  ICD-10 diagnosis: T86.1xx-T86.4xx, T86.81x, T86.85x, T86.89x, T86.9xx, Y83.0x, Z48.2xx (except for Z48.290), Z94.0x-Z94.4x, Z94.82, Z94.83, Z94.89, Z94.9x  ICD-10 procedure: 02YAxxx, 0BYCxxx-0BYMxxx, 0DY5xxx, 0DY6xxx, 0DY8xxx, 0DYExxx, 0FSGxxx, 0FY0xxx, 0FYGxxx, 0TY0xxx, 0TY1xxx, 3E030Ux, 3E033Ux, 3E0J3Ux, 3E0J7Ux, 3E0J8Ux  CPT/HCPCS: 32851-32854, 33935, 33945, 44135, 44136, 47135, 47136, 48554, 48556, 50360, 50365, 50370, 50380 | Any setting, any position |
| HIV/AIDS | ICD-9 DX: 042.xx, 079.53, V08.xx  ICD-10 DX: B20.xxx, B97.35x, Z21.xxx | Any setting, any position |
| Cancer | ICD-9 DX: 140.xx-172.xx, 174.xx-208.xx, 209.0x-209.3x, 207.xx  ICD-10 DX: C00.xxx-C43.xxx, C4A.xxx, C45.xxx-C96.xxx, D45.xxx | Any setting, any position |

# Supplemental Table 2. Outcome definitions

| **Outcome** | **Components** | **Diagnosis and/or Procedure Codes** | **Setting/Position** | |
| --- | --- | --- | --- | --- |
| MACE | MI | ICD-9 diagnosis: 410.x  ICD-10 diagnosis: I21.x (excluding I21.9, I21.Ax) | Inpatient, primary or secondary position | |
|  | Stroke | ICD-9 diagnosis: 430, 431, 433.x1, 434.x1, 436  ICD-10 diagnosis: I60.x, I61.x, I63.x, I67.89 | Inpatient, primary position | |
|  | CV mortality | ***Medicare***  NDI ICD-10 Cause of CV Death Code: I00.x - I99.x | Primary cause of death | |
| Composite of MI or stroke | MI | Same definition reported for the MACE outcome |  | |
|  | Stroke | Same definition reported for the MACE outcome |  | |
| Hospitalized Heart Failure (HHF) | -- | ICD-9 diagnosis: 428.xx, 398.91, 402.x1, 404.x1, 404.x3  ICD-10 diagnosis: I09.81, I11.0, I13.0, I13.2, I50.xxx | Inpatient, primary position | |
| End-stage kidney disease (including dialysis or renal transplant) |  | ICD-9 diagnosis: 585.5, 585.6, 996.81, V42.0, V45.1x, V56.xx  ICD-9 procedure: 39.95, 54.98, 55.6x  ICD-10 diagnosis: N18.5, N18.6, R88.0, T82.41x, T82.42x, T82.43x, T82.49x, T85.611x, T85.621x, T85.631x, T85.71x, T86.1x, Y84.1, Z48.22, Z49.xx, Z91.15, Z94.0, Z99.2  ICD-10 procedure: 0TY00Zx, 0TY10Zx, 3E1M39Z, 5A1Dx0Z  HCPCS/CPT: 50360, 50365, 90920, 90921, 90924, 90925, 90935, 90937, 90939, 90940, 90945, 90947, 90957, 90958, 90959, 90960, 90961, 90962, 90965, 90966, 90969, 90970, 90989, 90993, 90999, 90997, 99512, 99559, 99512, G0257, G0314, G0315, G0316, G0317, G0318, G0319, G0322, G0323, G0326, G0327, S9335, S9339 | Any setting, any position | |
| All-cause mortality | | ***Medicare***  Vital Status File & NDI ICD-10 Cause of Death when available |  |  |
| Coronary revascularization | | ICD-9 procedure: 00.66, 36.03, 36.06, 36.07, 36.09, 36.1x, 36.2x, 36.3x  ICD-10 procedure: 0210.xxx, 0211.xxx, 0212.xxx, 0213.xxx, 021K0Z5, 021K4Z5, 021L0Z5, 021L4Z5, 0270.xxx, 0271.xxx, 0272.xxx, 0273.xxx, 02C0.xxx, 02C1.xxx, 02C2.xxx, 02C3.xxx, 02QA.xxx, 02QB.xxx, 02QC.xxx  CPT/HCPCS: 33140, 33141, 33510-33536, 33545, 33572, 92920, 92921, 92924, 92925, 92928, 92929, 92933, 92934, 92937, 92938, 92941, 92943, 92944, 92973, 92980, 92981, 92984, 92995, 92996 | Inpatient, any position |  |
| Unstable angina hospitalization | | ICD-9 diagnosis: 411.1, 411.8x  ICD-10 diagnosis: I20.0, I24.8, I24.9, I25.110, I25.7x0 | Inpatient, any position |  |

# Supplemental Table 3. Baseline characteristics of patients initiating empagliflozin or GLP-1RA before and after 1:1 PS matching

|  | | **Before 1:1 Matching** | | | **After 1:1 Matching** | | |
| --- | --- | --- | --- | --- | --- | --- | --- |
|  | | **Empagliflozin** | **GLP-1RA** | **St. Diff** | **Empagliflozin** | **GLP-1RA** | **St. Diff** |
| Number of patients | | 169,599 | 298,298 |  | N=141,541 | N=141,541 |  |
| Cohort Entry Year | |  |  |  |  |  |  |
| ...2014; n (%) | | 2,424 (1.4%) | 12,449 (4.2%) | 0.1703 | 2,305 (1.6%) | 2,444 (1.7%) | 0.0079 |
| ...2015; n (%) | | 11,743 (6.9%) | 37,496 (12.6%) | 0.1930 | 10,913 (7.7%) | 10,852 (7.7%) | 0.0000 |
| ...2016; n (%) | | 20,246 (11.9%) | 48,025 (16.1%) | 0.1213 | 18,290 (12.9%) | 18,008 (12.7%) | -0.0060 |
| ...2017; n (%) | | 38,596 (22.8%) | 59,642 (20.0%) | -0.0683 | 32,231 (22.8%) | 32,386 (22.9%) | 0.0024 |
| ...2018; n (%) | | 39,485 (23.3%) | 70,666 (23.7%) | 0.0094 | 34,398 (24.3%) | 34,179 (24.1%) | -0.0047 |
| ...2019; n (%) | | 57,105 (33.7%) | 70,020 (23.5%) | -0.2272 | 43,404 (30.7%) | 43,672 (30.9%) | 0.0043 |
| Age, years [mean (SD)] | | 63.10 (8.62) | 62.34 (8.71) | -0.0877 | 62.57 (8.64) | 62.62 (8.65) | 0.0058 |
| Age categories, years | |  |  |  |  |  |  |
| ...18-39; n (%) | | 5,981 (3.5%) | 14,204 (4.8%) | 0.0652 | 5,511 (3.9%) | 5,514 (3.9%) | 0.0000 |
| ...40 - 44; n (%) | | 7,083 (4.2%) | 14,249 (4.8%) | 0.0289 | 6,343 (4.5%) | 6,257 (4.4%) | -0.0048 |
| ...45 - 49; n (%) | | 11,788 (7.0%) | 21,981 (7.4%) | 0.0155 | 10,408 (7.4%) | 10,467 (7.4%) | 0.0000 |
| ...50 - 54; n (%) | | 16,192 (9.5%) | 28,119 (9.4%) | -0.0034 | 13,988 (9.9%) | 14,037 (9.9%) | 0.0000 |
| ...55 - 59; n (%) | | 19,567 (11.5%) | 31,799 (10.7%) | -0.0255 | 16,613 (11.7%) | 16,283 (11.5%) | -0.0062 |
| ...60 - 64; n (%) | | 19,098 (11.3%) | 29,437 (9.9%) | -0.0455 | 15,773 (11.1%) | 15,452 (10.9%) | -0.0064 |
| ...65 - 69; n (%) | | 33,313 (19.6%) | 65,863 (22.1%) | 0.0616 | 28,064 (19.8%) | 28,579 (20.2%) | 0.0100 |
| ...70 - 74; n (%) | | 30,036 (17.7%) | 52,063 (17.5%) | -0.0053 | 24,506 (17.3%) | 24,638 (17.4%) | 0.0026 |
| ...75 - 79; n (%) | | 16,732 (9.9%) | 26,427 (8.9%) | -0.0343 | 12,935 (9.1%) | 13,023 (9.2%) | 0.0035 |
| ...80 - 84; n (%) | | 6,929 (4.1%) | 10,072 (3.4%) | -0.0369 | 5,232 (3.7%) | 5,147 (3.6%) | -0.0053 |
| ...>= 85; n (%) | | 2,880 (1.7%) | 4,084 (1.4%) | -0.0243 | 2,168 (1.5%) | 2,144 (1.5%) | 0.0000 |
| Gender - Female; n (%) | | 73,320 (43.2%) | 162,186 (54.4%) | 0.2255 | 65,824 (46.5%) | 65,369 (46.2%) | -0.0060 |
| Geographic region | |  |  |  |  |  |  |
| ...Northeast; n (%) | | 26,334 (15.5%) | 42,612 (14.3%) | -0.0337 | 21,147 (14.9%) | 21,184 (15.0%) | 0.0028 |
| ...South; n (%) | | 84,139 (49.6%) | 147,155 (49.3%) | -0.0060 | 70,845 (50.1%) | 70,735 (50.0%) | -0.0020 |
| ...Midwest; n (%) | | 31,641 (18.7%) | 63,154 (21.2%) | 0.0626 | 27,601 (19.5%) | 27,504 (19.4%) | -0.0025 |
| ...West; n (%) | | 27,485 (16.2%) | 45,377 (15.2%) | -0.0275 | 21,948 (15.5%) | 22,118 (15.6%) | 0.0028 |
| Race categories* | |  |  |  |  |  |  |
| ...White; n (%) | | 78,748 (70.6%) | 145,670 (75.1%) | 0.1013 | 66,543 (72.9%) | 66,387 (72.7%) | -0.0045 |
| ...Black; n (%) | | 10,932 (9.8%) | 20,744 (10.7%) | 0.0297 | 9,330 (10.2%) | 9,313 (10.2%) | 0.0000 |
| ...Asian; n (%) | | 5,637 (5.1%) | 4,872 (2.5%) | -0.1363 | 3,176 (3.5%) | 3,253 (3.6%) | 0.0054 |
| ...Hispanic; n (%) | | 10,501 (9.4%) | 14,957 (7.7%) | -0.0608 | 8,152 (8.9%) | 8,141 (8.9%) | 0.0000 |
| ...Other or unknown; n (%) | | 5,663 (5.1%) | 7,814 (4.0%) | -0.0528 | 4,134 (4.5%) | 4,241 (4.6%) | 0.0048 |
| Combined comorbidity score^†^ [mean (SD)] | | 1.16 (1.66) | 1.40 (1.76) | 0.1403 | 1.17 (1.66) | 1.19 (1.63) | 0.0122 |
| Frailty Score [mean (SD)] | | 0.16 (0.04) | 0.17 (0.04) | 0.2500 | 0.16 (0.04) | 0.16 (0.04) | 0.0000 |
| Frailty Score: (Categories) | |  |  |  |  |  |  |
| ...0.00 - 0.14; n (%) | | 86,816 (51.2%) | 131,656 (44.1%) | -0.1425 | 71,262 (50.3%) | 70,610 (49.9%) | -0.0080 |
| ...0.15 - 0.24; n (%) | | 73,601 (43.4%) | 141,785 (47.5%) | 0.0824 | 62,079 (43.9%) | 62,781 (44.4%) | 0.0101 |
| ...>= 0.25; n (%) | | 9,182 (5.4%) | 24,857 (8.3%) | 0.1150 | 8,200 (5.8%) | 8,150 (5.8%) | 0.0000 |
| Baseline CVD [sub-group]; n (%) | | 58,858 (34.7%) | 98,937 (33.2%) | -0.0317 | 46,906 (33.1%) | 46,904 (33.1%) | 0.0000 |
| Overweight; n (%) | | 17,385 (10.3%) | 22,350 (7.5%) | -0.0985 | 12,868 (9.1%) | 12,948 (9.1%) | 0.0000 |
| Obesity; n (%) | | 62,936 (37.1%) | 142,318 (47.7%) | 0.2157 | 57,224 (40.4%) | 57,072 (40.3%) | -0.0020 |
| Smoking; n (%) | | 30,693 (18.1%) | 57,606 (19.3%) | 0.0308 | 25,682 (18.1%) | 25,745 (18.2%) | 0.0026 |
| Alcohol abuse or dependence; n (%) | | 1,908 (1.1%) | 2,993 (1.0%) | -0.0098 | 1,492 (1.1%) | 1,558 (1.1%) | 0.0000 |
| Drug abuse or dependence; n (%) | | 2,678 (1.6%) | 5,850 (2.0%) | 0.0301 | 2,337 (1.7%) | 2,388 (1.7%) | 0.0000 |
| Diabetic nephropathy; n (%) | | 21,758 (12.8%) | 52,661 (17.7%) | 0.1366 | 19,041 (13.5%) | 19,333 (13.7%) | 0.0058 |
| Diabetic retinopathy; n (%) | | 17,559 (10.4%) | 36,210 (12.1%) | 0.0538 | 14,902 (10.5%) | 14,956 (10.6%) | 0.0033 |
| Diabetes with other ophthalmic manifestations; n (%) | | 10,657 (6.3%) | 21,389 (7.2%) | 0.0359 | 8,778 (6.2%) | 8,706 (6.2%) | 0.0000 |
| Diabetic neuropathy; n (%) | | 35,709 (21.1%) | 76,604 (25.7%) | 0.1088 | 31,042 (21.9%) | 31,485 (22.2%) | 0.0072 |
| Diabetes with peripheral circulatory disorders; n (%) | | 1,031 (0.6%) | 4,032 (1.4%) | 0.0805 | 957 (0.7%) | 970 (0.7%) | 0.0000 |
| Diabetic foot; n (%) | | 3,596 (2.1%) | 9,246 (3.1%) | 0.0629 | 3,263 (2.3%) | 3,269 (2.3%) | 0.0000 |
| Infection of lower extremities (cellulitis or osteomyelitis) ; n (%) | | 6,874 (4.1%) | 15,377 (5.2%) | 0.0523 | 6,046 (4.3%) | 6,041 (4.3%) | 0.0000 |
| Lower-limb amputations; n (%) | | 802 (0.5%) | 2,052 (0.7%) | 0.0259 | 720 (0.5%) | 684 (0.5%) | 0.0000 |
| Erectile dysfunction; n (%) | | 8,424 (5.0%) | 12,269 (4.1%) | -0.0432 | 6,683 (4.7%) | 6,798 (4.8%) | 0.0047 |
| Hypoglycemia; n (%) | | 18,263 (10.8%) | 33,505 (11.2%) | 0.0128 | 15,028 (10.6%) | 15,186 (10.7%) | 0.0032 |
| Hyperglycemia; n (%) | | 83,573 (49.3%) | 149,606 (50.2%) | 0.0180 | 70,774 (50.0%) | 71,025 (50.2%) | 0.0040 |
| Diabetic ketoacidosis; n (%) | | 541 (0.3%) | 1,387 (0.5%) | 0.0317 | 478 (0.3%) | 494 (0.3%) | 0.0000 |
| Hyperosmolar hyperglycemic nonketotic syndrome (HONK); n (%) | | 1,473 (0.9%) | 2,893 (1.0%) | 0.0103 | 1,246 (0.9%) | 1,240 (0.9%) | 0.0000 |
| Diabetes with other complications; n (%) | | 17,925 (10.6%) | 35,161 (11.8%) | 0.0381 | 15,320 (10.8%) | 15,266 (10.8%) | 0.0000 |
| Diabetes mellitus without mention of complications; n (%) | | 151,777 (89.5%) | 269,435 (90.3%) | 0.0266 | 126,648 (89.5%) | 126,789 (89.6%) | 0.0033 |
| No. of glucose-lowering medications on cohort entry^§^ [mean (SD)] | | 1.47 (0.94) | 1.38 (0.95) | -0.0952 | 1.43 (0.94) | 1.44 (0.95) | 0.0106 |
| No use of anti-DM medications in prior 365 days; n (%) | | 10,500 (6.2%) | 20,504 (6.9%) | 0.0283 | 8,420 (5.9%) | 8,227 (5.8%) | -0.0043 |
| Initiation of empagliflozin or GLP-1RA monotherapy; n (%) | | 6,358 (3.7%) | 17,209 (5.8%) | 0.0988 | 6,058 (4.3%) | 5,770 (4.1%) | -0.0100 |
| DUAL therapy with metformin; n (%) | | 25,233 (14.9%) | 33,713 (11.3%) | -0.1069 | 20,361 (14.4%) | 20,123 (14.2%) | -0.0057 |
| Metformin (any use); n (%) | | 139,424 (82.2%) | 220,334 (73.9%) | -0.2015 | 114,420 (80.8%) | 114,619 (81.0%) | 0.0051 |
| Metformin (concurrent use); n (%) | | 111,898 (66.0%) | 168,128 (56.4%) | -0.1980 | 90,281 (63.8%) | 90,587 (64.0%) | 0.0042 |
| Metformin (past use); n (%) | | 27,526 (16.2%) | 52,206 (17.5%) | 0.0347 | 24,139 (17.1%) | 24,032 (17.0%) | -0.0027 |
| Sulfonylureas - 2nd generation (any use); n (%) | | 67,731 (39.9%) | 116,640 (39.1%) | -0.0164 | 57,505 (40.6%) | 57,294 (40.5%) | -0.0020 |
| Sulfonylureas - 2nd generation (concurrent use); n (%) | | 50,679 (29.9%) | 85,236 (28.6%) | -0.0286 | 42,935 (30.3%) | 42,806 (30.2%) | -0.0022 |
| Sulfonylureas - 2nd generation (past use); n (%) | | 17,052 (10.1%) | 31,404 (10.5%) | 0.0132 | 14,570 (10.3%) | 14,488 (10.2%) | -0.0033 |
| Thiazolidinediones (TZD) (any use); n (%) | | 15,423 (9.1%) | 26,796 (9.0%) | -0.0035 | 13,086 (9.2%) | 13,101 (9.3%) | 0.0035 |
| Thiazolidinediones (TZD) (concurrent use); n (%) | | 10,642 (6.3%) | 18,250 (6.1%) | -0.0083 | 9,035 (6.4%) | 9,037 (6.4%) | 0.0000 |
| Thiazolidinediones (TZD) (past use); n (%) | | 4,781 (2.8%) | 8,546 (2.9%) | 0.0060 | 4,051 (2.9%) | 4,064 (2.9%) | 0.0000 |
| DPP4i (any use); n (%) | | 62,785 (37.0%) | 82,214 (27.6%) | -0.2020 | 47,299 (33.4%) | 47,962 (33.9%) | 0.0106 |
| DPP4i (concurrent use); n (%) | | 48,223 (28.4%) | 50,477 (16.9%) | -0.2774 | 33,916 (24.0%) | 34,633 (24.5%) | 0.0117 |
| DPP4i (past use); n (%) | | 14,562 (8.6%) | 31,737 (10.6%) | 0.0679 | 13,383 (9.5%) | 13,329 (9.4%) | -0.0034 |
| Insulins (any use); n (%) | | 34,945 (20.6%) | 113,210 (38.0%) | 0.3895 | 33,966 (24.0%) | 34,650 (24.5%) | 0.0117 |
| Insulins (concurrent use); n (%) | | 25,703 (15.2%) | 86,114 (28.9%) | 0.3351 | 25,053 (17.7%) | 25,656 (18.1%) | 0.0104 |
| Insulins (past use); n (%) | | 9,242 (5.4%) | 27,097 (9.1%) | 0.1430 | 8,913 (6.3%) | 8,995 (6.4%) | 0.0041 |
| Long term use of insulin; n (%) | | 22,375 (13.2%) | 66,946 (22.4%) | 0.2423 | 21,229 (15.0%) | 21,715 (15.3%) | 0.0084 |
| Miscellaneous anti-DM medications; n (%) | | 3,349 (2.0%) | 6,637 (2.2%) | 0.0139 | 2,863 (2.0%) | 2,885 (2.0%) | 0.0000 |
| Hypertension; n (%) | | 136,850 (80.7%) | 243,774 (81.7%) | 0.0256 | 113,968 (80.5%) | 114,130 (80.6%) | 0.0025 |
| Hyperlipidemia; n (%) | | 135,953 (80.2%) | 236,375 (79.2%) | -0.0249 | 112,462 (79.5%) | 112,816 (79.7%) | 0.0050 |
| Acute MI; n (%) | | 4,118 (2.4%) | 5,315 (1.8%) | -0.0419 | 2,901 (2.0%) | 2,901 (2.0%) | 0.0000 |
| MI sequelae/old MI^‡^; n (%) | | 7,564 (4.5%) | 11,519 (3.9%) | -0.0299 | 5,687 (4.0%) | 5,730 (4.0%) | 0.0000 |
| Stable angina; n (%) | | 10,982 (6.5%) | 15,160 (5.1%) | -0.0599 | 8,081 (5.7%) | 8,059 (5.7%) | 0.0000 |
| Unstable angina; n (%) | | 5,173 (3.1%) | 7,080 (2.4%) | -0.0428 | 3,713 (2.6%) | 3,715 (2.6%) | 0.0000 |
| Coronary atherosclerosis; n (%) | | 41,041 (24.2%) | 63,563 (21.3%) | -0.0692 | 31,543 (22.3%) | 31,692 (22.4%) | 0.0024 |
| Coronary procedure; n (%) | | 4,663 (2.7%) | 5,050 (1.7%) | -0.0682 | 3,014 (2.1%) | 3,029 (2.1%) | 0.0000 |
| History of coronary procedure; n (%) | | 15,582 (9.2%) | 22,157 (7.4%) | -0.0653 | 11,387 (8.0%) | 11,449 (8.1%) | 0.0037 |
| Heart failure; n (%) | | 14,415 (8.5%) | 28,221 (9.5%) | 0.0349 | 11,649 (8.2%) | 11,771 (8.3%) | 0.0036 |
| Cardiomyopathy; n (%) | | 5,660 (3.3%) | 9,496 (3.2%) | -0.0056 | 4,471 (3.2%) | 4,487 (3.2%) | 0.0000 |
| Atrial fibrillation; n (%) | | 13,643 (8.0%) | 23,407 (7.8%) | -0.0074 | 10,869 (7.7%) | 11,016 (7.8%) | 0.0037 |
| Cardiac conduction disorders; n (%) | | 7,392 (4.4%) | 12,633 (4.2%) | -0.0099 | 5,812 (4.1%) | 5,881 (4.2%) | 0.0050 |
| Other cardiac dysrhythmia; n (%) | | 18,173 (10.7%) | 31,464 (10.5%) | -0.0065 | 14,556 (10.3%) | 14,566 (10.3%) | 0.0000 |
| Valve disorders; n (%) | | 16,035 (9.5%) | 26,521 (8.9%) | -0.0208 | 12,556 (8.9%) | 12,665 (8.9%) | 0.0000 |
| Other cardiovascular disease; n (%) | | 21,775 (12.8%) | 37,341 (12.5%) | -0.0090 | 17,532 (12.4%) | 17,677 (12.5%) | 0.0030 |
| Ischemic stroke; n (%) | | 13,366 (7.9%) | 22,506 (7.5%) | -0.0150 | 10,683 (7.5%) | 10,653 (7.5%) | 0.0000 |
| TIA; n (%) | | 3,145 (1.9%) | 5,424 (1.8%) | -0.0074 | 2,521 (1.8%) | 2,603 (1.8%) | 0.0000 |
| Other cerebrovascular conditions; n (%) | | 8,362 (4.9%) | 15,943 (5.3%) | 0.0182 | 6,898 (4.9%) | 6,867 (4.9%) | 0.0000 |
| PAD and generalized/unspecified atherosclerosis; n (%) | | 14,605 (8.6%) | 27,478 (9.2%) | 0.0211 | 12,117 (8.6%) | 12,072 (8.5%) | -0.0036 |
| Acute Kidney Injury; n (%) | | 4,426 (2.6%) | 13,057 (4.4%) | 0.0981 | 3,975 (2.8%) | 4,067 (2.9%) | 0.0060 |
| CKD; n (%) | | 16,430 (9.7%) | 49,998 (16.8%) | 0.2106 | 15,022 (10.6%) | 15,522 (11.0%) | 0.0129 |
| CKD Stages 1-2; n (%) | | 6,139 (3.6%) | 12,264 (4.1%) | 0.0260 | 5,087 (3.6%) | 5,173 (3.7%) | 0.0053 |
| CKD Stages 3-4; n (%) | | 11,652 (6.9%) | 40,195 (13.5%) | 0.2194 | 11,001 (7.8%) | 11,261 (8.0%) | 0.0074 |
| CKD unspecified; n (%) | | 4,874 (2.9%) | 17,781 (6.0%) | 0.1508 | 4,985 (3.5%) | 5,132 (3.6%) | 0.0054 |
| Hypertensive nephropathy; n (%) | | 9,260 (5.5%) | 27,869 (9.3%) | 0.1455 | 8,340 (5.9%) | 8,491 (6.0%) | 0.0042 |
| Proteinuria; n (%) | | 8,360 (4.9%) | 19,230 (6.4%) | 0.0650 | 7,241 (5.1%) | 7,295 (5.2%) | 0.0045 |
| Miscellaneous renal disease; n (%) | | 11,267 (6.6%) | 27,552 (9.2%) | 0.0965 | 9,817 (6.9%) | 10,001 (7.1%) | 0.0078 |
| UTI; n (%) | | 17,241 (10.2%) | 40,562 (13.6%) | 0.1052 | 15,258 (10.8%) | 15,386 (10.9%) | 0.0032 |
| Kidney and urinary stone; n (%) | | 6,929 (4.1%) | 13,374 (4.5%) | 0.0197 | 5,862 (4.1%) | 5,984 (4.2%) | 0.0050 |
| Disorders of electrolyte; n (%) | | 9,714 (5.7%) | 21,438 (7.2%) | 0.0611 | 8,242 (5.8%) | 8,350 (5.9%) | 0.0043 |
| Disorders of fluid balance; n (%) | | 4,718 (2.8%) | 10,430 (3.5%) | 0.0401 | 3,985 (2.8%) | 3,989 (2.8%) | 0.0000 |
| Edema; n (%) | | 14,590 (8.6%) | 35,379 (11.9%) | 0.1090 | 12,879 (9.1%) | 13,160 (9.3%) | 0.0069 |
| COPD; n (%) | | 13,986 (8.2%) | 28,471 (9.5%) | 0.0458 | 11,842 (8.4%) | 11,958 (8.4%) | 0.0000 |
| Asthma; n (%) | | 13,065 (7.7%) | 28,918 (9.7%) | 0.0710 | 11,489 (8.1%) | 11,424 (8.1%) | 0.0000 |
| Obstructive sleep apnea; n (%) | | 29,185 (17.2%) | 66,566 (22.3%) | 0.1284 | 26,246 (18.5%) | 26,285 (18.6%) | 0.0026 |
| Pneumonia; n (%) | | 5,726 (3.4%) | 12,128 (4.1%) | 0.0369 | 4,906 (3.5%) | 4,969 (3.5%) | 0.0000 |
| Osteoarthritis; n (%) | | 35,963 (21.2%) | 72,591 (24.3%) | 0.0740 | 31,052 (21.9%) | 31,150 (22.0%) | 0.0024 |
| Osteoporosis without fractures; n (%) | | 7,030 (4.1%) | 12,871 (4.3%) | 0.0100 | 5,744 (4.1%) | 5,716 (4.0%) | -0.0051 |
| Fractures; n (%) | | 2,521 (1.5%) | 5,352 (1.8%) | 0.0236 | 2,199 (1.6%) | 2,213 (1.6%) | 0.0000 |
| Falls; n (%) | | 6,250 (3.7%) | 12,845 (4.3%) | 0.0306 | 5,388 (3.8%) | 5,421 (3.8%) | 0.0000 |
| Hypothyroidism; n (%) | | 31,319 (18.5%) | 64,767 (21.7%) | 0.0799 | 27,210 (19.2%) | 27,297 (19.3%) | 0.0025 |
| Hyperthyroidism and other thyroid gland disorders; n (%) | | 13,424 (7.9%) | 26,579 (8.9%) | 0.0361 | 11,516 (8.1%) | 11,538 (8.2%) | 0.0037 |
| NASH/NAFLD; n (%) | | 10,549 (6.2%) | 19,469 (6.5%) | 0.0123 | 9,095 (6.4%) | 9,161 (6.5%) | 0.0041 |
| Liver disease; n (%) | | 5,979 (3.5%) | 10,293 (3.5%) | 0.0000 | 4,871 (3.4%) | 4,865 (3.4%) | 0.0000 |
| Depression; n (%) | | 22,084 (13.0%) | 53,373 (17.9%) | 0.1359 | 20,031 (14.2%) | 20,068 (14.2%) | 0.0000 |
| Anxiety and sleep disorders; n (%) | | 31,472 (18.6%) | 66,371 (22.2%) | 0.0894 | 27,773 (19.6%) | 27,590 (19.5%) | -0.0025 |
| Dementia; n (%) | | 4,114 (2.4%) | 8,159 (2.7%) | 0.0190 | 3,460 (2.4%) | 3,469 (2.5%) | 0.0065 |
| Psychosis; n (%) | | 1,017 (0.6%) | 2,257 (0.8%) | 0.0240 | 895 (0.6%) | 827 (0.6%) | 0.0000 |
| Delirium; n (%) | | 1,073 (0.6%) | 2,419 (0.8%) | 0.0240 | 898 (0.6%) | 979 (0.7%) | 0.0124 |
| ACEi and ARBs; n (%) | | 126,421 (74.5%) | 222,629 (74.6%) | 0.0023 | 105,435 (74.5%) | 105,697 (74.7%) | 0.0046 |
| Beta blockers; n (%) | | 64,467 (38.0%) | 114,431 (38.4%) | 0.0082 | 52,505 (37.1%) | 52,849 (37.3%) | 0.0041 |
| Calcium channel blockers - DHP & Non-DHP; n (%) | | 47,395 (27.9%) | 85,226 (28.6%) | 0.0155 | 39,151 (27.7%) | 39,242 (27.7%) | 0.0000 |
| Nitrates and other antianginal agents; n (%) | | 12,237 (7.2%) | 20,071 (6.7%) | -0.0197 | 9,449 (6.7%) | 9,560 (6.8%) | 0.0040 |
| Thiazide and thiazide-like diuretics; n (%) | | 22,002 (13.0%) | 45,473 (15.2%) | 0.0632 | 19,162 (13.5%) | 19,134 (13.5%) | 0.0000 |
| Loop diuretics; n (%) | | 19,517 (11.5%) | 49,735 (16.7%) | 0.1498 | 17,285 (12.2%) | 17,625 (12.5%) | 0.0091 |
| MRA (potassium-sparing diuretics); n (%) | | 6,729 (4.0%) | 14,863 (5.0%) | 0.0483 | 5,750 (4.1%) | 5,743 (4.1%) | 0.0000 |
| Other K-sparing diuretics and antihypertensive medications; n (%) | | 12,384 (7.3%) | 27,614 (9.3%) | 0.0725 | 10,842 (7.7%) | 10,801 (7.6%) | -0.0038 |
| Digoxin; n (%) | | 2,358 (1.4%) | 3,812 (1.3%) | -0.0087 | 1,826 (1.3%) | 1,853 (1.3%) | 0.0000 |
| Entresto; n (%) | | 987 (0.6%) | 889 (0.3%) | -0.0448 | 700 (0.5%) | 514 (0.4%) | -0.0149 |
| Antiarrhythmics; n (%) | | 2,819 (1.7%) | 4,989 (1.7%) | 0.0000 | 2,277 (1.6%) | 2,317 (1.6%) | 0.0000 |
| Anticoagulants (oral); n (%) | | 11,921 (7.0%) | 21,884 (7.3%) | 0.0116 | 9,674 (6.8%) | 9,734 (6.9%) | 0.0040 |
| Anticoagulants (injectables); n (%) | | 663 (0.4%) | 1,659 (0.6%) | 0.0284 | 567 (0.4%) | 597 (0.4%) | 0.0000 |
| Antiplatelet agents; n (%) | | 20,880 (12.3%) | 31,380 (10.5%) | -0.0567 | 15,921 (11.2%) | 16,072 (11.4%) | 0.0063 |
| Statins; n (%) | | 127,087 (74.9%) | 214,951 (72.1%) | -0.0635 | 104,200 (73.6%) | 104,425 (73.8%) | 0.0045 |
| PCSK9 inhibitors and other-lipid lowering agents; n (%) | | 27,069 (16.0%) | 46,642 (15.6%) | -0.0110 | 21,962 (15.5%) | 22,251 (15.7%) | 0.0055 |
| COPD & asthma medications; n (%) | | 29,879 (17.6%) | 62,379 (20.9%) | 0.0838 | 25,973 (18.4%) | 26,258 (18.6%) | 0.0052 |
| Corticosteroids (oral); n (%) | | 29,454 (17.4%) | 57,153 (19.2%) | 0.0466 | 25,378 (17.9%) | 25,507 (18.0%) | 0.0026 |
| Antiosteoporosis agents; n (%) | | 4,683 (2.8%) | 7,810 (2.6%) | -0.0123 | 3,662 (2.6%) | 3,633 (2.6%) | 0.0000 |
| NSAIDs; n (%) | | 45,663 (26.9%) | 85,231 (28.6%) | 0.0380 | 38,873 (27.5%) | 39,039 (27.6%) | 0.0022 |
| Opioids; n (%) | | 49,528 (29.2%) | 105,449 (35.4%) | 0.1329 | 43,272 (30.6%) | 43,662 (30.8%) | 0.0043 |
| Gabapentinoids; n (%) | | 24,700 (14.6%) | 56,688 (19.0%) | 0.1179 | 21,981 (15.5%) | 22,212 (15.7%) | 0.0055 |
| UTI antibiotics; n (%) | | 31,579 (18.6%) | 68,589 (23.0%) | 0.1086 | 27,747 (19.6%) | 27,902 (19.7%) | 0.0025 |
| Antidepressants; n (%) | | 42,821 (25.2%) | 99,949 (33.5%) | 0.1830 | 38,909 (27.5%) | 39,036 (27.6%) | 0.0022 |
| Anxiolytics/hypnotics; n (%) | | 11,932 (7.0%) | 25,596 (8.6%) | 0.0597 | 10,420 (7.4%) | 10,460 (7.4%) | 0.0000 |
| Benzodiazepines; n (%) | | 19,516 (11.5%) | 41,415 (13.9%) | 0.0721 | 17,160 (12.1%) | 17,166 (12.1%) | 0.0000 |
| Antipsychotics; n (%) | | 3,718 (2.2%) | 8,768 (2.9%) | 0.0444 | 3,327 (2.4%) | 3,456 (2.4%) | 0.0000 |
| Antiparkinsonian medications; n (%) | | 4,156 (2.5%) | 10,311 (3.5%) | 0.0586 | 3,738 (2.6%) | 3,768 (2.7%) | 0.0062 |
| Dementia medications; n (%) | | 2,399 (1.4%) | 4,691 (1.6%) | 0.0165 | 2,011 (1.4%) | 2,002 (1.4%) | 0.0000 |
| Internist (-365 days to CED); n (%) | | 152,108 (89.7%) | 267,999 (89.8%) | 0.0033 | 126,883 (89.6%) | 126,938 (89.7%) | 0.0033 |
| Internist (-30 days to CED); n (%) | | 110,466 (65.1%) | 187,744 (62.9%) | -0.0458 | 91,622 (64.7%) | 91,748 (64.8%) | 0.0021 |
| Internist (-365 days to -31 days before CED); n (%) | | 145,134 (85.6%) | 257,746 (86.4%) | 0.0231 | 121,391 (85.8%) | 121,278 (85.7%) | -0.0029 |
| No. of internist visits [mean (SD)] | | 14.28 (17.57) | 14.84 (18.88) | 0.0307 | 14.26 (17.39) | 14.41 (18.62) | 0.0083 |
| Endocrinologist (-365 days to CED); n (%) | | 32,401 (19.1%) | 73,922 (24.8%) | 0.1380 | 28,434 (20.1%) | 29,080 (20.5%) | 0.0099 |
| Endocrinologist (-30 days to CED); n (%) | | 23,709 (14.0%) | 54,114 (18.1%) | 0.1119 | 20,990 (14.8%) | 21,223 (15.0%) | 0.0056 |
| Endocrinologist (-365 days to -31 days before CED); n (%) | | 23,983 (14.1%) | 53,237 (17.8%) | 0.1012 | 21,053 (14.9%) | 20,649 (14.6%) | -0.0085 |
| No. of endocrinologist visits [mean (SD)] | | 1.43 (5.55) | 1.82 (6.18) | 0.0664 | 1.51 (5.68) | 1.52 (5.94) | 0.0017 |
| Cardiologist (-365 days to CED); n (%) | | 61,949 (36.5%) | 106,532 (35.7%) | -0.0167 | 49,734 (35.1%) | 49,702 (35.1%) | 0.0000 |
| Cardiologist (-30 days to CED); n (%) | | 19,830 (11.7%) | 26,889 (9.0%) | -0.0887 | 14,264 (10.1%) | 14,379 (10.2%) | 0.0033 |
| Cardiologist (-365 days to -31 days before CED); n (%) | | 57,662 (34.0%) | 100,309 (33.6%) | -0.0085 | 46,544 (32.9%) | 46,403 (32.8%) | -0.0021 |
| No. of cardiologist visits [mean (SD)] | | 2.90 (6.64) | 2.58 (6.22) | -0.0497 | 2.64 (6.18) | 2.65 (6.62) | 0.0016 |
| Nephrologist (-365 days to CED); n (%) | | 5,961 (3.5%) | 22,261 (7.5%) | 0.1761 | 5,591 (4.0%) | 5,788 (4.1%) | 0.0051 |
| Nephrologist (-30 days to CED); n (%) | | 1,542 (0.9%) | 5,760 (1.9%) | 0.0852 | 1,438 (1.0%) | 1,586 (1.1%) | 0.0098 |
| Nephrologist (-365 days to -31 days before CED); n (%) | | 5,501 (3.2%) | 20,860 (7.0%) | 0.1734 | 5,176 (3.7%) | 5,314 (3.8%) | 0.0053 |
| No. of nephrologist visits [mean (SD)] | | 0.17 (1.68) | 0.39 (2.57) | 0.1013 | 0.19 (1.73) | 0.21 (1.91) | 0.0110 |
| Electrocardiogram; n (%) | | 74,698 (44.0%) | 128,783 (43.2%) | -0.0161 | 60,850 (43.0%) | 60,281 (42.6%) | -0.0081 |
| No. of electrocardiogram tests [mean (SD)] | | 1.15 (2.10) | 1.12 (2.03) | -0.0145 | 1.11 (2.03) | 1.11 (2.06) | 0.0000 |
| ECG & Other cardiac imaging; n (%) | | 38,775 (22.9%) | 66,351 (22.2%) | -0.0168 | 30,855 (21.8%) | 30,983 (21.9%) | 0.0024 |
| No. of ECG & Other cardiac imaging [mean (SD)] | | 0.70 (2.33) | 0.61 (1.89) | -0.0424 | 0.64 (2.15) | 0.63 (2.05) | -0.0048 |
| Cardiovascular stress test; n (%) | | 19,467 (11.5%) | 32,754 (11.0%) | -0.0158 | 15,461 (10.9%) | 15,368 (10.9%) | 0.0000 |
| HbA1c test order; n (%) | | 159,244 (93.9%) | 278,984 (93.5%) | -0.0165 | 132,938 (93.9%) | 132,544 (93.6%) | -0.0124 |
| No. of HbA1c test orders [mean (SD)] | | 2.58 (1.39) | 2.63 (1.43) | 0.0355 | 2.59 (1.39) | 2.60 (1.42) | 0.0071 |
| Glucose test and monitoring; n (%) | | 54,346 (32.0%) | 106,146 (35.6%) | 0.0762 | 46,179 (32.6%) | 46,549 (32.9%) | 0.0064 |
| No. of glucose tests and monitoring [mean (SD)] | | 0.82 (2.11) | 1.04 (6.33) | 0.0466 | 0.84 (2.16) | 0.86 (2.80) | 0.0080 |
| Microalbuminuria/proteinuria test order; n (%) | | 106,532 (62.8%) | 186,249 (62.4%) | -0.0083 | 88,845 (62.8%) | 87,930 (62.1%) | -0.0145 |
| No. of microalbuminuria/ proteinuria test orders [mean (SD)] | | 0.97 (1.03) | 1.00 (1.10) | 0.0282 | 0.97 (1.02) | 0.97 (1.06) | 0.0000 |
| Metabolic or renal/creatinine panel test order; n (%) | | 149,774 (88.3%) | 263,006 (88.2%) | -0.0031 | 124,713 (88.1%) | 124,296 (87.8%) | -0.0092 |
| No. of metabolic or renal/creatinine panel test orders [mean (SD)] | | 2.71 (2.18) | 2.90 (2.41) | 0.0827 | 2.71 (2.20) | 2.74 (2.26) | 0.0135 |
| Lipid test order; n (%) | | 149,044 (87.9%) | 256,301 (85.9%) | -0.0593 | 123,818 (87.5%) | 122,984 (86.9%) | -0.0180 |
| No. of lipid test orders [mean (SD)] | | 2.01 (1.72) | 1.92 (1.74) | -0.0520 | 1.97 (1.71) | 1.97 (1.82) | 0.0000 |
| Uric acid test order; n (%) | | 18,648 (11.0%) | 35,070 (11.8%) | 0.0252 | 15,264 (10.8%) | 15,310 (10.8%) | 0.0000 |
| Vitamin D test order; n (%) | | 47,504 (28.0%) | 91,978 (30.8%) | 0.0615 | 39,913 (28.2%) | 40,645 (28.7%) | 0.0111 |
| PTH test order; n (%) | | 7,037 (4.1%) | 22,184 (7.4%) | 0.1421 | 6,284 (4.4%) | 7,259 (5.1%) | 0.0329 |
| No. of hospitalizations [mean (SD)] | | 0.14 (0.46) | 0.15 (0.48) | 0.0213 | 0.13 (0.45) | 0.13 (0.45) | 0.0000 |
| LOS (-365 days to -31 days before cohort entry) [mean (SD)] | | 0.56 (2.82) | 0.65 (3.08) | 0.0305 | 0.55 (2.60) | 0.55 (2.85) | 0.0000 |
| LOS (-30 days to cohort entry) [mean (SD)] | | 0.09 (0.89) | 0.07 (0.76) | -0.0242 | 0.08 (0.82) | 0.09 (0.78) | 0.0125 |
| No. of emergency visits [mean (SD)] | | 0.43 (1.23) | 0.50 (1.31) | 0.0551 | 0.44 (1.24) | 0.45 (1.26) | 0.0080 |
| No. of distinct medications [mean (SD)] | | 12.36 (5.62) | 13.86 (6.08) | 0.2562 | 12.63 (5.68) | 12.71 (5.55) | 0.0142 |
| No. of distinct brand medications [mean (SD)] | | 2.80 (1.70) | 3.21 (1.93) | 0.2254 | 2.85 (1.72) | 2.88 (1.76) | 0.0172 |
| Total Cost ($)^\|\|^  [mean (SD)] | | 12673.01 (19444.57) | 14503.33 (21962.77) | 0.0882 | 12661.83 (19214.09) | 13009.92 (19004.00) | 0.0182 |
| Inpatient Cost ($)^\|\|^  [mean (SD)] | | 2517.05 (12495.90) | 2450.98 (12222.94) | -0.0053 | 2335.39 (12030.60) | 2374.36 (12336.41) | 0.0032 |
| Outpatient Cost ($)^\|\|^  [mean (SD)] | | 3292.33 (8564.58) | 3600.64 (8979.10) | 0.0351 | 3324.15 (8482.47) | 3357.04 (8290.19) | 0.0039 |
| Pharmacy cost for glucose-lowering medications ($)^\|\|^  [mean (SD)] | | 2910.36 (3819.55) | 3819.13 (4565.23) | 0.2159 | 2971.61 (3975.42) | 3221.39 (3621.75) | 0.0657 |
| Pharmacy cost for non- glucose-lowering medications ($)^\|\|^  [mean (SD)] | | 2558.11 (7637.94) | 2976.17 (12096.76) | 0.0413 | 2604.63 (7766.19) | 2616.46 (7717.01) | 0.0015 |
| Out of pocket pharmacy cost ($) [mean (SD)] | | 730.06 (780.06) | 814.76 (866.16) | 0.1028 | 740.42 (790.32) | 752.85 (789.26) | 0.0157 |
| Home health agency cost^\|\|^  ($) [mean (SD)] | | 349.66 (2023.69) | 620.38 (3165.25) | 0.1019 | 353.72 (2035.38) | 586.30 (3078.64) | 0.0891 |
| Flu vaccine; n (%) | | 73,915 (43.6%) | 134,496 (45.1%) | 0.0302 | 61,251 (43.3%) | 62,397 (44.1%) | 0.0161 |
| Pneumoccocal vaccine; n (%) | | 23,318 (13.7%) | 43,753 (14.7%) | 0.0287 | 19,493 (13.8%) | 19,897 (14.1%) | 0.0087 |
| Breast mammography or MRI; n (%) | | 34,956 (20.6%) | 75,998 (25.5%) | 0.1165 | 31,476 (22.2%) | 31,760 (22.4%) | 0.0048 |
| Prostate DRE or PSA; n (%) | | 45,903 (27.1%) | 60,282 (20.2%) | -0.1629 | 35,294 (24.9%) | 34,814 (24.6%) | -0.0070 |
| Colonoscopy; n (%) | | 15,695 (9.3%) | 27,954 (9.4%) | 0.0034 | 13,220 (9.3%) | 13,037 (9.2%) | -0.0035 |
| Pap smear; n (%) | | 11,720 (6.9%) | 26,795 (9.0%) | 0.0777 | 10,711 (7.6%) | 10,770 (7.6%) | 0.0000 |
| Bone mineral density; n (%) | | 9,363 (5.5%) | 18,644 (6.3%) | 0.0340 | 8,041 (5.7%) | 8,171 (5.8%) | 0.0043 |
| *Laboratory results^#^* | |  |  |  |  |  |  |
| Hba1c, mean (SD) | | 9.0(2.31) | 9.0(2.41) | 0.016 | 9.0(2.31) | 9.0(2.39) | 0.001 |
| Creatinine, mean (SD) | | 1.0(0.47) | 1.0(0.50) | 0.077 | 1.0(0.47) | 1.0(0.47) | 0.059 |
| eGFR, mean (SD) | | 84.4(21.95) | 81.6(24.92) | 0.119 | 84.5(22.31) | 83.0(23.33) | 0.066 |
| UACR, mean (SD) | | 107.8(520.79) | 154.2(610.36) | 0.082 | 112.1(548.33) | 126.7(536.22) | 0.027 |
| Proteinuria, mean (SD) | | 866.2(1645.87) | 1099.2(2183.37) | 0.121 | 834.1(1654.90) | 919.1(1770.14) | 0.05 |
| Total cholesterol, mean (SD) | | 180.9(54.37) | 182.9(53.46) | 0.037 | 182.0(54.38) | 180.9(53.40) | 0.021 |
| LDL, mean (SD) | | 98.7(44.15) | 99.9(43.34) | 0.029 | 99.5(44.38) | 98.5(43.16) | 0.023 |
| HDL, mean (SD) | | 46.9(16.87) | 47.0(17.09) | 0.007 | 46.8(16.68) | 46.5(16.73) | 0.018 |
| Triglyceride, mean (SD) | | 206.2(163.88) | 206.1(156.88) | 0.001 | 208.2(165.01) | 206.8(158.86) | 0.009 |
| ACEI: angiotensin converting enzyme inhibitors; ARB: angiotensin receptor blockers; BB: beta blockers; CCB: calcium channel blockers; CED: cohort entry date; CKD: chronic kidney disease; COPD: chronic obstructive pulmonary diseases; CVD: cardiovascular disease; DM: diabetes mellitus; ED: emergency department; DPP4i: dipeptidyl peptidase-4 inhibitors; GLP-1RA: glucagon-like peptide-1 receptor agonist; LOS: length of stay; MI: myocardial infarction; NASH: non-alcoholic steatohepatitis; NAFLD: non-alcoholic fatty liver disease; PAD: peripheral arterial diseases; PCSK9: proprotein convertase subtilisin/kexin type 9 serine protease; PS: propensity score; PSA: prostate surface antigen; SD: standard deviation; SGLT2i: sodium-glucose cotransporter 2 inhibitors; SMD: standardized mean difference; SU: sulfonylurea; TZD: thiazolidinediones.  Baseline characteristics were measured during 12 months prior to and including the index date (cohort entry date) unless otherwise stated.  * Race information is only available in Medicare and Clinformatics® and not reported in Marketscan.  † Calculated using the weights in Gagne JJ, Glynn RJ, Avorn J, Levin R, Schneeweiss S. A combined comorbidity score predicted mortality in elderly patients better than existing scores. J Clin Epidemiol. 2011 Jul;64(7):749-59. doi: 10.1016/j.jclinepi.2010.10.004. Epub 2011 Jan 5. PMID: 21208778; PMCID: PMC3100405.  ‡ Represents the structural and functional complications of myocardial infarction. Defined using ICD-9-CM and ICD-10-CM diagnosis codes.  § Number of diabetes medications calculated here did not include the index medications.  \|\| Includes deductibles, copayments, and coinsurance.  # Laboratory results were available in Clinformatics® (approximately 45%) and MarketScan (approximately 5-10%) databases, thus not included in the PS model. | | | | | | | |
|  | | | | | | | |

# Supplemental Table 4. Baseline characteristics of patients initiating empagliflozin or GLP-1RA among patients with non-missing laboratory results data after 1:1 PS matching

| **Characteristic** | **Empagliflozin N=20,334** | **GLP-1RA N=20,334** | **Total N=40,668** | **Standardized Differences** |
| --- | --- | --- | --- | --- |
| Age, mean(SD) | 59.2(11.64) | 59.2(11.65) | 59.2(11.65) | 0.001 |
| Female | 9,355 (46.0%) | 9,404 (46.2%) | 18,759 (46.1%) | 0.005 |
| Combined comorbidity score, mean(SD) ^*^ | 1.3(1.93) | 1.3(1.94) | 1.3(1.93) | 0.008 |
| Frailty index, mean(SD) | 0.1(0.04) | 0.1(0.04) | 0.1(0.04) | 0.016 |
| HbA1c, mean(SD) | 9.0(2.27) | 9.0(2.27) | 9.0(2.27) | 0.005 |
| Creatinine, mean(SD) | 1.0(0.35) | 1.0(0.33) | 1.0(0.34) | 0.013 |
| UACR, mean(SD) | 100.4(438.97) | 108.3(449.25) | 104.4(444.15) | 0.018 |
| Total cholesterol, mean(SD) | 181.0(52.38) | 179.3(50.25) | 180.2(51.33) | 0.032 |
| LDL, mean(SD) | 98.3(42.09) | 97.1(40.69) | 97.7(41.40) | 0.030 |
| HDL, mean(SD) | 46.8(15.93) | 46.0(15.39) | 46.4(15.67) | 0.051 |
| Triglyceride, mean(SD) | 204.0(157.90) | 204.1(152.95) | 204.1(155.45) | 0.000 |
| Glucose, mean(SD) | 189.8(76.88) | 190.0(76.06) | 189.9(76.47) | 0.004 |
| eGFR, mean(SD) | 84.7(22.00) | 84.8(21.96) | 84.8(21.98) | 0.004 |
| Diabetes nephropathy | 3,182 (15.6%) | 3,193 (15.7%) | 6,375 (15.7%) | 0.001 |
| Diabetes retinopathy | 1,974 (9.7%) | 1,971 (9.7%) | 3,945 (9.7%) | 0.000 |
| Diabetes ophthalmopathy | 1,068 (5.3%) | 1,126 (5.5%) | 2,194 (5.4%) | 0.013 |
| Diabetes neuropathy | 4,472 (22.0%) | 4,495 (22.1%) | 8,967 (22.0%) | 0.003 |
| Hypoglycemia | 3,072 (15.1%) | 3,072 (15.1%) | 6,144 (15.1%) | 0.000 |
| Hyperglycemia | 10,806 (53.1%) | 10,805 (53.1%) | 21,611 (53.1%) | 0.000 |
| Metformin use (current) | 12,997 (63.9%) | 13,089 (64.4%) | 26,086 (64.1%) | 0.009 |
| Sulfonylurea use (current) | 5,917 (29.1%) | 5,937 (29.2%) | 11,854 (29.1%) | 0.002 |
| DPP-4i use (current) | 4,331 (21.3%) | 4,368 (21.5%) | 8,699 (21.4%) | 0.004 |
| Insulin use (current) | 3,234 (15.9%) | 3,251 (16.0%) | 6,485 (15.9%) | 0.002 |
| MI | 312 (1.5%) | 304 (1.5%) | 616 (1.5%) | 0.003 |
| MI sequelae^†^ | 687 (3.4%) | 666 (3.3%) | 1,353 (3.3%) | 0.006 |
| Stable angina | 1,055 (5.2%) | 1,022 (5.0%) | 2,077 (5.1%) | 0.007 |
| Unstable angina | 436 (2.1%) | 414 (2.0%) | 850 (2.1%) | 0.008 |
| Coronary atherosclerosis | 3,374 (16.6%) | 3,403 (16.7%) | 6,777 (16.7%) | 0.004 |
| Coronary procedure | 338 (1.7%) | 320 (1.6%) | 658 (1.6%) | 0.007 |
| History of coronary procedure | 1,157 (5.7%) | 1,119 (5.5%) | 2,276 (5.6%) | 0.008 |
| Heart failure | 1,270 (6.2%) | 1,304 (6.4%) | 2,574 (6.3%) | 0.007 |
| Cardiomyopathy | 559 (2.7%) | 564 (2.8%) | 1,123 (2.8%) | 0.002 |
| Atrial fibrillation | 995 (4.9%) | 1,035 (5.1%) | 2,030 (5.0%) | 0.009 |
| Stroke | 1,042 (5.1%) | 1,027 (5.1%) | 2,069 (5.1%) | 0.003 |
| TIA | 277 (1.4%) | 286 (1.4%) | 563 (1.4%) | 0.004 |
| Peripheral arterial disease | 1,514 (7.4%) | 1,511 (7.4%) | 3,025 (7.4%) | 0.001 |
| Acute kidney injury | 443 (2.2%) | 446 (2.2%) | 889 (2.2%) | 0.001 |
| CKD stages 1-2 | 1,063 (5.2%) | 1,106 (5.4%) | 2,169 (5.3%) | 0.009 |
| CKD stages 3-4 | 1,530 (7.5%) | 1,500 (7.4%) | 3,030 (7.5%) | 0.006 |
| CKD unknown stage | 526 (2.6%) | 543 (2.7%) | 1,069 (2.6%) | 0.005 |
| COPD | 1,448 (7.1%) | 1,494 (7.3%) | 2,942 (7.2%) | 0.009 |
| Asthma | 1,596 (7.8%) | 1,658 (8.2%) | 3,254 (8.0%) | 0.011 |
| Sleep apnea | 3,461 (17.0%) | 3,484 (17.1%) | 6,945 (17.1%) | 0.003 |
| Pneumonia | 560 (2.8%) | 579 (2.8%) | 1,139 (2.8%) | 0.006 |
| Osteoarthritis | 3,554 (17.5%) | 3,644 (17.9%) | 7,198 (17.7%) | 0.012 |
| Osteoporosis | 657 (3.2%) | 658 (3.2%) | 1,315 (3.2%) | 0.000 |
| Fractures | 218 (1.1%) | 209 (1.0%) | 427 (1.0%) | 0.004 |
| Falls | 550 (2.7%) | 569 (2.8%) | 1,119 (2.8%) | 0.006 |
| Non-alcoholic fatty liver disease | 1,472 (7.2%) | 1,455 (7.2%) | 2,927 (7.2%) | 0.003 |
| Liver disease | 764 (3.8%) | 769 (3.8%) | 1,533 (3.8%) | 0.001 |
| Depression | 2,828 (13.9%) | 2,854 (14.0%) | 5,682 (14.0%) | 0.004 |
| Anxiety | 3,775 (18.6%) | 3,790 (18.6%) | 7,565 (18.6%) | 0.002 |
| Dementia | 283 (1.4%) | 298 (1.5%) | 581 (1.4%) | 0.006 |
| ACEI | 14,921 (73.4%) | 15,006 (73.8%) | 29,927 (73.6%) | 0.009 |
| Beta-blockers | 6,475 (31.8%) | 6,513 (32.0%) | 12,988 (31.9%) | 0.004 |
| CCB | 5,290 (26.0%) | 5,306 (26.1%) | 10,596 (26.1%) | 0.002 |
| Nitrates | 999 (4.9%) | 981 (4.8%) | 1,980 (4.9%) | 0.004 |
| Thiazides | 2,472 (12.2%) | 2,490 (12.2%) | 4,962 (12.2%) | 0.003 |
| Loop diuretics | 1,812 (8.9%) | 1,865 (9.2%) | 3,677 (9.0%) | 0.009 |
| Mineralocorticoid receptor antagonists | 649 (3.2%) | 673 (3.3%) | 1,322 (3.3%) | 0.007 |
| Other potassium-sparing | 1,401 (6.9%) | 1,411 (6.9%) | 2,812 (6.9%) | 0.002 |
| Digoxin | 175 (0.9%) | 176 (0.9%) | 351 (0.9%) | 0.001 |
| Antiarrhythmics | 216 (1.1%) | 221 (1.1%) | 437 (1.1%) | 0.002 |
| Anticoagulants | 923 (4.5%) | 956 (4.7%) | 1,879 (4.6%) | 0.008 |
| Antiplatelets | 2,026 (10.0%) | 2,086 (10.3%) | 4,112 (10.1%) | 0.010 |
| Statins | 14,739 (72.5%) | 14,819 (72.9%) | 29,558 (72.7%) | 0.009 |
| PCSK-9 inhibitors | 3,060 (15.0%) | 3,137 (15.4%) | 6,197 (15.2%) | 0.011 |
| COPD medications | 3,459 (17.0%) | 3,496 (17.2%) | 6,955 (17.1%) | 0.005 |
| Steroids | 3,477 (17.1%) | 3,528 (17.4%) | 7,005 (17.2%) | 0.007 |
| Antiosteoporosis | 457 (2.2%) | 455 (2.2%) | 912 (2.2%) | 0.001 |
| NSAIDs | 5,836 (28.7%) | 5,827 (28.7%) | 11,663 (28.7%) | 0.001 |
| Opioids | 5,969 (29.4%) | 6,021 (29.6%) | 11,990 (29.5%) | 0.006 |
| Antidementia medications | 194 (1.0%) | 180 (0.9%) | 374 (0.9%) | 0.007 |
| ACEI: angiotensin converting enzyme inhibitors; ARB: angiotensin receptor blockers; CKD: chronic kidney disease; COPD: chronic obstructive pulmonary disease; DM: diabetes mellitus; EMPA: empagliflozin; GLP-1RA: glucagon like peptide-1 receptor agonists; HbA1c: hemoglobin A1c; MI: myocardial infarction; PCSK9: proprotein convertase subtilisin/kexin type 9 serine protease; PS: propensity score; SD: standard deviation; St. Diff: absolute standardized mean differences (<0.1 was suggested as a measure of satisfactory balance as in Austin PC. Assessing covariate balance when using the generalized propensity score with quantitative or continuous exposures. Stat Methods Med Res. 2019 May;28(5):1365-1377. doi: 10.1177/0962280218756159. Epub 2018 Feb 8. PMID: 29415624; PMCID: PMC6484705.).  Baseline characteristics were measured during 12 months prior to and including the index date (cohort entry date) unless otherwise stated.  * Calculated using the weights in Gagne JJ, Glynn RJ, Avorn J, Levin R, Schneeweiss S. A combined comorbidity score predicted mortality in elderly patients better than existing scores. J Clin Epidemiol. 2011 Jul;64(7):749-59. doi: 10.1016/j.jclinepi.2010.10.004. Epub 2011 Jan 5. PMID: 21208778; PMCID: PMC3100405.  † Represents the structural and functional complications of myocardial infarction. Defined using ICD-9-CM and ICD-10-CM diagnosis codes. | | | | |

# Supplemental Table 5. Follow-up Time and Censoring Reason for Primary Outcomes Between 1:1 PS-Matched Initiators of Empagliflozin vs GLP-1RA*

| **Overall population** | **Overall**  **N = 283,082** | **Empagliflozin**  **N = 141,541** | **GLP-1RA**  **N = 141,541** |
| --- | --- | --- | --- |
| **MI and stroke** |  |  |  |
| Follow-up, mean (SD), days | 253 (253) | 259 (258) | 246 (248) |
| Follow-up, median (25^th^, 75^th^ IQR), days | 158 (88, 321) | 164 (88, 332) | 152 (88, 311) |
| Censoring reasons (%): |  |  |  |
| Treatment discontinuation | 40.2% | 39.4% | 41.0% |
| End of study - 9/30/2019 | 34.9% | 35.4% | 34.3% |
| Treatment switching | 10.9% | 10.8% | 11.1% |
| Disenrollment | 12.6% | 13.1% | 12.2% |
| Outcome development | 0.9% | 0.9% | 0.9% |
| All-cause mortality | 0.4% | 0.2% | 0.2% |
| **HHF outcome** |  |  |  |
| Follow-up, mean (SD), days | 254 (254) | 260 (260) | 247 (248) |
| Follow-up, median (25^th^, 75^th^ IQR), days | 158 (88, 323) | 165 (88, 334) | 153 (88, 312) |
| Censoring reasons (%): |  |  |  |
| Treatment discontinuation | 40.4% | 39.6% | 41.2% |
| End of study - 9/30/2019 | 35.0% | 35.6% | 34.5% |
| Treatment switching | 10.9% | 10.8% | 11.2% |
| Disenrollment | 12.7% | 13.2% | 12.2% |
| Outcome development | 0.4% | 0.4% | 0.5% |
| All-cause mortality | 0.5% | 0.5% | 0.5% |
| **MACE** |  |  |  |
| Follow-up, mean (SD), days | 251 (251) | 254 (251) | 247 (251) |
| Follow-up, median (25^th^, 75^th^ IQR), days | 153 (88, 307) | 158 (88, 318) | 150 (88, 297) |
| Censoring reasons (%): |  |  |  |
| Treatment discontinuation | 44.5% | 43.8% | 45.1% |
| End of study - 9/30/2019 | 39.8% | 40.4% | 39.2% |
| Treatment switching | 8.8% | 8.8% | 8.9% |
| Disenrollment | 4.8% | 4.9% | 4.6% |
| Outcome development | 1.6% | 1.6% | 1.7% |
| Non-cardiovascular mortality | 0.5% | 0.5% | 0.6% |
| **Cardiovascular death and HHF outcome** |  |  |  |
| Follow-up, mean (SD), days | 252 (252) | 255 (252) | 248 (252) |
| Follow-up, median (25^th^, 75^th^ IQR), days | 155 (88, 309) | 159 (88, 320) | 150 (88, 298) |
| Censoring reasons (%): |  |  |  |
| Treatment discontinuation | 44.7% | 44.1% | 45.4% |
| End of study - 9/30/2019 | 39.9% | 40.7% | 39.3% |
| Treatment switching | 8.9% | 8.8% | 8.9% |
| Disenrollment | 4.8% | 4.9% | 4.6% |
| Outcome development | 1.1% | 1.0% | 1.2% |
| Non-cardiovascular mortality | 0.6% | 0.5% | 0.6% |

HHF: hospitalization for heart failure; IQR: interquartile range; MACE: major adverse cardiovascular events; PS: propensity-score; SD: standard deviation;

* Censoring was outcome-specific. The table reports follow-up and censoring information specific to the primary outcomes. Censoring reasons specific to other study outcomes may include minor variations.

# Supplemental Table 6. Database-specific estimates for primary effectiveness outcomes between 1:1 PS-matched initiators of empagliflozin and GLP-1RA

|  |  | Empagliflozin | GLP-1RA | Empagliflozin vs. GLP-1RA | |
| --- | --- | --- | --- | --- | --- |
| Primary outcomes | No. of matched pairs | N events  (IR/1,000 PY) | N events  (IR/1,000 PY) | HR  (95% CI) | RD/1,000PY  (95% CI) |
| **Medicare** |  |  |  |  |  |
| Composite of myocardial infarction or stroke | 54,292 | 710 (18.79) | 737 (20.1) | 0.93 (0.84, 1.04) | -1.31 (-3.31, 0.70) |
| Hospitalization for heart failure | 54,292 | 366 (9.64) | 482 (13.09) | 0.73 (0.64, 0.84) | -3.45 (-4.99, -1.93) |
| MACE outcome* | 54,292 | 854 (22.6) | 922 (25.14) | 0.90 (0.82, 0.99) | -2.54 (-4.76, -0.32) |
| Composite of cardiovascular death or hospitalization for heart failure* | 54,292 | 540 (14.22) | 675 (18.34) | 0.77 (0.69, 0.86) | -4.11 (-5.95, -2.29) |
| **Optum** |  |  |  |  |  |
| Composite of myocardial infarction or stroke | 37,043 | 301 (11.82) | 257 (11) | 1.09 (0.92, 1.28) | 0.83 (-1.08, 2.72) |
| Hospitalization for heart failure | 37,043 | 87 (3.4) | 131 (5.59) | 0.61 (0.47, 0.80) | -2.19 (-3.41, -1.01) |
| **Marketscan** |  |  |  |  |  |
| Composite of myocardial infarction or stroke | 50,206 | 306 (8.25) | 280 (7.92) | 1.04 (0.89, 1.23) | 0.33 (-0.98, 1.64) |
| Hospitalization for heart failure | 50,206 | 54 (1.45) | 87 (2.45) | 0.59 (0.42, 0.83) | -1.00 (-1.66, -0.37) |
| CI: confidence intervals; HR: hazard ratio; IR: Incidence rate; MACE: major adverse cardiovascular events; PS: propensity score; PY: person-years; RD: rate difference.  * MACE outcome (which includes hospitalization for myocardial infarction, or ischemic or hemorrhagic stroke, and cardiovascular-specific mortality), and cardiovascular-specific (or all-cause) mortality data were only available in Medicare database, the only database where mortality data was completely and reliably reported and the data linkage with National Death Index was possible.  ^†^ Defined using diagnosis codes in inpatient setting, with any diagnosis fields on hospital discharge, following a validated algorithm shown to have high specificity and positive predictive value.  ^‡^ Defined using diagnosis codes in inpatient setting, with primary diagnosis field on hospital discharge, following a validated algorithm shown to have high specificity and positive predictive value. | | | | | |

# Supplemental Table 7. Subgroup analyses for secondary outcomes pooled across 3 databases

|  |  | **Empagliflozin** | **GLP-1RA** | **Empagliflozin vs. GLP-1RA** | |
| --- | --- | --- | --- | --- | --- |
| **Secondary outcomes** | **No. of matched pairs** | **N events**  **(IR/1,000 PY)** | **N events**  **(IR/1,000 PY)** | **HR**  **(95% CI)** | **RD/1,000PY**  **(95% CI)** |
| **Composite of MI, stroke, or HHF** |  |  |  |  |  |
| Age≥65 years | 72,602 | 1301 (26.07) | 1454 (30.08) | 0.86 (0.80, 0.93) | -4.00 (-6.10, -1.91) |
| Age<65 years | 69,101 | 475 (9.38) | 440 (9.31) | 1.01 (0.89, 1.15) | 0.07 (-1.14, 1.28) |
| Male | 76,326 | 1054 (18.46) | 1132 (21.58) | 0.86 (0.79, 0.94) | -3.12 (-4.80, -1.44) |
| Female | 65,659 | 719 (16.44) | 776 (17.93) | 0.92 (0.83, 1.01) | -1.49 (-3.24, 0.25) |
| ASCVD | 44,158 | 1117 (37.24) | 1273 (45.12) | 0.83 (0.77, 0.90) | -7.88 (-11.19, -4.58) |
| No ASCVD | 97,430 | 640 (9.12) | 672 (10.03) | 0.91 (0.82, 1.02) | -0.91 (-1.95, 0.13) |
| HF | 11,435 | 515 (73.19) | 628 (92.86) | 0.79 (0.71, 0.89) | -19.67 (-29.33, -10.06) |
| No HF | 130,037 | 1212 (13.03) | 1250 (14.13) | 0.93 (0.86, 1.00) | -1.10 (-2.17, -0.03) |
| **Composite of MACE or HHF (older Medicare patients only)** |  |  |  |  |  |
| Male | 27,665 | 658 (32.46) | 731 (38.48) | 0.84 (0.76, 0.93) | -6.03 (-9.77, -2.30) |
| Female | 26,677 | 497 (28.51) | 538 (31.13) | 0.92 (0.81, 1.04) | -2.62 (-6.26, 1.01) |
| ASCVD | 25,930 | 821 (47.02) | 946 (56.97) | 0.83 (0.76, 0.91) | -9.95 (-14.81, -5.11) |
| No ASCVD | 28,374 | 337 (16.70) | 361 (18.21) | 0.92 (0.79, 1.06) | -1.51 (-4.10, 1.08) |
| HF | 7,430 | 421 (92.19) | 493 (111.44) | 0.83 (0.73, 0.94) | -19.24 (-32.50, -6.06) |
| No HF | 46,824 | 718 (21.74) | 761 (23.80) | 0.91 (0.83, 1.01) | -2.06 (-4.39, 0.26) |
| **Hospitalization for heart failure (broad)** |  |  |  |  |  |
| Age≥65 years | 72,602 | 1936 (39.01) | 2149 (44.77) | 0.87 (0.82, 0.93) | -5.76 (-8.33, -3.19) |
| Age<65 years | 69,101 | 373 (7.36) | 431 (9.12) | 0.81 (0.70, 0.93) | -1.76 (-2.91, -0.62) |
| Male | 76,326 | 1398 (24.54) | 1452 (27.77) | 0.90 (0.83, 0.96) | -3.23 (-5.16, -1.31) |
| Female | 65,659 | 948 (21.71) | 1104 (25.59) | 0.85 (0.78, 0.92) | -3.88 (-5.93, -1.83) |
| ASCVD | 44,158 | 1708 (57.45) | 1870 (66.95) | 0.87 (0.82, 0.93) | -9.51 (-13.59, -5.43) |
| No ASCVD | 97,430 | 609 (8.68) | 723 (10.8) | 0.81 (0.73, 0.91) | -2.13 (-3.18, -1.08) |
| HF | 11,435 | 1336 (201.03) | 1387 (215.95) | 0.94 (0.87, 1.01) | -14.92 (-30.60, 0.73) |
| No HF | 130,037 | 949 (10.18) | 1196 (13.52) | 0.76 (0.70, 0.83) | -3.33 (-4.34, -2.33) |
| **Myocardial infarction** |  |  |  |  |  |
| Age≥65 years | 72,602 | 537 (10.69) | 579 (11.88) | 0.90 (0.80, 1.01) | -1.19 (-2.52, 0.13) |
| Age<65 years | 69,101 | 297 (5.86) | 246 (5.2) | 1.13 (0.95, 1.34) | 0.66 (-0.27, 1.59) |
| Male | 76,326 | 514 (8.97) | 526 (9.98) | 0.91 (0.80, 1.02) | -1.01 (-2.16, 0.14) |
| Female | 65,659 | 292 (6.65) | 309 (7.11) | 0.93 (0.80, 1.10) | -0.46 (-1.56, 0.64) |
| ASCVD | 44,158 | 485 (16.03) | 516 (18.09) | 0.90 (0.79, 1.01) | -2.06 (-4.18, 0.05) |
| No ASCVD | 97,430 | 328 (4.67) | 299 (4.45) | 1.05 (0.90, 1.23) | 0.21 (-0.50, 0.93) |
| HF | 11,435 | 163 (22.73) | 198 (28.56) | 0.80 (0.65, 0.98) | -5.83 (-11.16, -0.55) |
| No HF | 130,037 | 643 (6.89) | 610 (6.87) | 1.01 (0.90, 1.13) | 0.02 (-0.74, 0.78) |
| **Stroke** |  |  |  |  |  |
| Age≥65 years | 72,602 | 409 (8.13) | 378 (7.74) | 1.05 (0.92, 1.21) | 0.40 (-0.72, 1.51) |
| Age<65 years | 69,101 | 110 (2.16) | 99 (2.09) | 1.05 (0.80, 1.38) | 0.08 (-0.50, 0.65) |
| Male | 76,326 | 281 (4.89) | 264 (4.99) | 1.00 (0.84, 1.18) | -0.10 (-0.94, 0.73) |
| Female | 65,659 | 244 (5.55) | 225 (5.17) | 1.07 (0.89, 1.28) | 0.38 (-0.59, 1.35) |
| ASCVD | 44,158 | 303 (9.98) | 294 (10.26) | 0.98 (0.84, 1.15) | -0.28 (-1.91, 1.35) |
| No ASCVD | 97,430 | 218 (3.1) | 226 (3.36) | 0.93 (0.77, 1.12) | -0.26 (-0.87, 0.34) |
| HF | 11,435 | 85 (11.8) | 84 (12.01) | 0.99 (0.73, 1.35) | -0.21 (-3.83, 3.40) |
| No HF | 130,037 | 422 (4.52) | 388 (4.37) | 1.05 (0.91, 1.20) | 0.15 (-0.46, 0.76) |
| **Cardiovascular mortality*** |  |  |  |  |  |
| Male | 27,665 | 122 (5.95) | 124 (6.42) | 0.92 (0.72, 1.18) | -0.48 (-2.04, 1.07) |
| Female | 26,677 | 78 (4.42) | 81 (4.63) | 0.96 (0.71, 1.32) | -0.21 (-1.63, 1.21) |
| ASCVD | 25,930 | 141 (7.93) | 169 (9.96) | 0.80 (0.64, 1.00) | -2.03 (-4.04, -0.04) |
| No ASCVD | 28,374 | 61 (3) | 62 (3.1) | 0.97 (0.68, 1.38) | -0.10 (-1.19, 0.99) |
| HF | 7,430 | 76 (16.14) | 88 (19.1) | 0.85 (0.63, 1.15) | -2.96 (-8.41, 2.44) |
| No HF | 46,824 | 123 (3.69) | 126 (3.9) | 0.94 (0.74, 1.21) | -0.21 (-1.16, 0.73) |
| **All-cause mortality** |  |  |  |  |  |
| Male | 27,665 | 302 (27665) | 296 (27665) | 0.95 (0.81, 1.12) | -0.61 (-3.03, 1.80) |
| Female | 26,677 | 213 (26677) | 227 (26677) | 0.93 (0.78, 1.13) | -0.90 (-3.25, 1.44) |
| ASCVD | 25,930 | 318 (25930) | 370 (25930) | 0.82 (0.70, 0.95) | -3.92 (-6.90, -0.95) |
| No ASCVD | 28,374 | 198 (28374) | 180 (28374) | 1.08 (0.89, 1.32) | 0.74 (-1.16, 2.64) |
| HF | 7,430 | 177 (7430) | 191 (7430) | 0.91 (0.74, 1.11) | -3.87 (-11.98, 4.21) |
| No HF | 46,824 | 336 (46824) | 338 (46824) | 0.96 (0.83, 1.12) | -0.39 (-1.94, 1.16) |
| **Unstable angina** |  |  |  |  |  |
| Age≥65 years | 72,602 | 171 (3.39) | 170 (3.47) | 0.98 (0.79, 1.21) | -0.08 (-0.81, 0.65) |
| Age<65 years | 69,101 | 96 (1.89) | 82 (1.73) | 1.08 (0.80, 1.45) | 0.16 (-0.38, 0.69) |
| Male | 76,326 | 176 (3.06) | 166 (3.14) | 0.98 (0.79, 1.21) | -0.08 (-0.74, 0.58) |
| Female | 65,659 | 97 (2.2) | 89 (2.04) | 1.08 (0.81, 1.43) | 0.16 (-0.45, 0.77) |
| ASCVD | 44,158 | 183 (6.02) | 180 (6.28) | 0.96 (0.79, 1.18) | -0.25 (-1.53, 1.01) |
| No ASCVD | 97,430 | 72 (1.02) | 67 (1) | 1.03 (0.73, 1.43) | 0.03 (-0.31, 0.36) |
| HF | 11,435 | 55 (7.63) | 42 (6) | 1.28 (0.86, 1.92) | 1.63 (-1.09, 4.39) |
| No HF | 130,037 | 209 (2.24) | 200 (2.25) | 1.00 (0.82, 1.21) | -0.01 (-0.45, 0.42) |
| **Coronary revascularization** |  |  |  |  |  |
| Age≥65 years | 72,602 | 654 (13.05) | 643 (13.21) | 0.99 (0.89, 1.10) | -0.16 (-1.59, 1.27) |
| Age<65 years | 69,101 | 347 (6.85) | 308 (6.51) | 1.05 (0.90, 1.22) | 0.34 (-0.69, 1.36) |
| Male | 76,326 | 689 (12.05) | 663 (12.59) | 0.97 (0.87, 1.08) | -0.54 (-1.86, 0.77) |
| Female | 65,659 | 319 (7.26) | 289 (6.65) | 1.09 (0.93, 1.28) | 0.62 (-0.49, 1.73) |
| ASCVD | 44,158 | 600 (19.89) | 601 (21.1) | 0.95 (0.85, 1.07) | -1.21 (-3.53, 1.11) |
| No ASCVD | 97,430 | 386 (5.5) | 318 (4.74) | 1.16 (1.00, 1.35) | 0.76 (0.00, 1.52) |
| HF | 11,435 | 156 (21.79) | 173 (24.88) | 0.88 (0.71, 1.09) | -3.10 (-8.17, 1.94) |
| No HF | 130,037 | 819 (8.79) | 774 (8.73) | 1.01 (0.92, 1.12) | 0.06 (-0.80, 0.92) |
| \| ASCVD: atherosclerotic cardiovascular disease; CI: confidence intervals; HF: heart failure; HR: hazard ratio; IR: Incidence rate; PS: propensity score; PY: person-years; RD: rate difference.  *Cardiovascular mortality outcome was available only in Medicare beneficiaries aged 65 years and older. \| \| --- \| | | | | | |

# Supplemental Table 8. Sensitivity analyses for 1:1 PS-matched initiators of empagliflozin vs. GLP-1RA

|  | |  | Empagliflozin | GLP-1RA | Empagliflozin vs. GLP-1RA | |
| --- | --- | --- | --- | --- | --- | --- |
| **Primary and secondary outcomes** | | **No. of matched pairs** | **N events**  **(IR/1,000 PY)** | **N events**  **(IR/1,000 PY)** | **HR**  **(95% CI)** | **RD/1,000PY**  **(95% CI)** |
| **Dual Metformin Therapy with No Insulin** | |  |  |  |  |  |
| Composite of myocardial infarction or stroke | | 40,423 | 239 (9.21) | 254 (10.6) | 0.87 (0.73, 1.04) | -1.39 (-3.15, 0.35) |
| Hospitalization for heart failure (HHF) | | 40,423 | 71 (2.73) | 102 (4.25) | 0.63 (0.47, 0.86) | -1.52 (-2.58, -0.49) |
| MACE outcome* | | 15,178 | 154 (15.84) | 163 (18.42) | 0.86 (0.69, 1.08) | -2.58 (-6.39, 1.18) |
| Composite of cardiovascular death or HHF* | | 15,178 | 77 (7.89) | 101 (11.37) | 0.68 (0.51, 0.92) | -3.48 (-6.37, -0.67) |
| **Grace period 30 days** | |  |  |  |  |  |
| Composite of myocardial infarction or stroke | | 141,541 | 1082 (12.96) | 1005 (13.09) | 1.00 (0.92, 1.09) | -0.13 (-1.25, 0.99) |
| Hospitalization for heart failure (HHF) | | 141,541 | 378 (4.51) | 515 (6.69) | 0.67 (0.59, 0.77) | -2.18 (-2.92, -1.45) |
| MACE outcome* | | 54,292 | 693 (22.04) | 707 (24.11) | 0.92 (0.82, 1.02) | -2.07 (-4.50, 0.34) |
| Composite of cardiovascular death or HHF* | | 54,292 | 415 (13.15) | 503 (17.1) | 0.76 (0.67, 0.87) | -3.96 (-5.92, -2.00) |
| **ITT 2 years** | |  |  |  |  |  |
| Composite of myocardial infarction or stroke | | 141,873 | 2235 (14.43) | 2331 (15.04) | 0.96 (0.91, 1.02) | -0.61 (-1.47, 0.24) |
| Hospitalization for heart failure (HHF) | | 141,873 | 1022 (6.56) | 1328 (8.53) | 0.77 (0.71, 0.83) | -1.97 (-2.58, -1.36) |
| MACE outcome* | | 54,371 | 1556 (25.53) | 1668 (27.42) | 0.93 (0.87, 1.00) | -1.89 (-3.72, -0.06) |
| Composite of cardiovascular death or HHF* | | 54,371 | 1054 (17.17) | 1255 (20.53) | 0.84 (0.77, 0.91) | -3.36 (-4.90, -1.82) |
| **Censoring-weighted analyses** | |  |  |  |  |  |
| Composite of myocardial infarction or stroke | | 141,541 | 1,351 (6.12) | 1,297 (6.19) | 1.01 (0.93, 1.10) | -0.06 (-0.53, 0.41) |
| Hospitalization for heart failure (HHF) | | 141,541 | 535 (2.42) | 725 (3.45) | 0.69 (0.62, 0.78) | -1.03 (-1.36, -0.71) |
| MACE outcome* | | 54,292 | 865 (10.38) | 925 (11.56) | 0.90 (0.82, 0.99) | -1.18 (-2.20, -0.16) |
| Composite of cardiovascular death or HHF* | | 54,292 | 546 (6.53) | 673 (8.38) | 0.76 (0.68, 0.85) | -1.85 (-2.69, -1.02) |
|  | CI: confidence intervals; HR: hazard ratio; IR: Incidence rate; MACE: major adverse cardiovascular events; PS: propensity score; PY: person-years; RD: rate differences.  * MACE outcome (which includes hospitalization for myocardial infarction, ischemic or hemorrhagic stroke, or all-cause mortality), and cardiovascular mortality data are only available for the Medicare database.  † Restricted to patients with chronic kidney disease stages 3-4 to allow progression of kidney disease within a short follow-up period available in our study. | | | | | |

# Supplemental Table 9. Analyses restricted to patients with non-missing laboratory result data for the propensity score matched initiators of empagliflozin vs. GLP-1RA with respect to primary cardiovascular effectiveness outcomes*

|  |  | **Empagliflozin** | **GLP-1RA** | **Empagliflozin vs. GLP-1RA** | |  |
| --- | --- | --- | --- | --- | --- | --- |
| **Primary and secondary outcomes** | **N of matched pairs*** | **N events**  **(IR/1,000 PY)** | **N events**  **(IR/1,000 PY)** | **HR**  **(95% CI)** | **RD/1,000PY**  **(95% CI)** |  |
| Composite of myocardial infarction and stroke^†^ | 20,334 | 145 (10.53) | 135 (9.96) | 1.06 (0.84, 1.34) | 0.57 (-1.84, 2.98) |  |
| Hospitalization for heart failure^†^ | 20,334 | 40 (2.89) | 62 (4.56) | 0.64 (0.43, 0.95) | -1.67 (-3.15, -0.24) |  |
| CI: confidence intervals; HR: hazard ratio; IR: Incidence rate; MACE: major adverse cardiovascular events; PS: propensity score; PY: person-years; RD: rate difference.  * Matching was performed using propensity score adjusting for 143 claims-based predictors plus HbA1c and eGFR. Analyses were restricted to 40,668 patients from Clinformatics and Marketscan with non-missing HbA1c and eGFR. eGFR was calculated using 2021 equation without race.  † Defined using diagnosis codes in inpatient settings, with primary diagnosis fields on hospital discharge, following a validated algorithm shown to have high specificity and positive predictive value. | | | | | | |

# Supplemental Table 10. High-dimensional propensity score matched initiators of empagliflozin vs. GLP-1RA with respect to primary cardiovascular effectiveness outcomes

|  |  | **Empagliflozin** | **GLP-1RA** | **Empagliflozin vs. GLP-1RA** | |  |
| --- | --- | --- | --- | --- | --- | --- |
| **Primary and secondary outcomes** | **N of matched pairs** | **N events**  **(IR/1,000 PY)** | **N events**  **(IR/1,000 PY)** | **HR**  **(95% CI)** | **RD/1,000PY**  **(95% CI)** |  |
| Composite of myocardial infarction or stroke | 137,570 | 1263 (12.93) | 1234 (13.36) | 0.98 (0.90, 1.06) | -0.42 (-1.46, 0.61) |  |
| Hospitalization for heart failure | 137,712 | 493 (5.02) | 701 (7.56) | 0.67 (0.59, 0.75) | -2.55 (-3.26, -1.84) |  |
| MACE outcome* | 52,268 | 812 (22.27) | 849 (24.29) | 0.92 (0.84, 1.01) | -2.02 (-4.26, 0.22) |  |
| Composite of cardiovascular death or hospitalization for heart failure* | 52,273 | 516 (14.08) | 657 (18.77) | 0.75 (0.67, 0.84) | -4.69 (-6.58, -2.82) |  |
| End-stage kidney disease | 10,837 | 128 (19.73) | 180 (26.5) | 0.75 (0.60, 0.94) | -6.77 (-11.97, -1.61) |  |
| CI: confidence intervals; HR: hazard ratio; IR: Incidence rate; MACE: major adverse cardiovascular events; PS: propensity score; PY: person-years; RD: rate difference.  * MACE outcome (which includes hospitalization for myocardial infarction, or ischemic or hemorrhagic stroke, all-cause mortality), and cardiovascular mortality data are only available for the Medicare database. | | | | | | |

# Supplemental Table 11. Analyses restricted to patients with at least one and two years of follow-up data with respect to primary cardiovascular effectiveness outcomes

|  |  | Empagliflozin | GLP-1RA | Empagliflozin vs. GLP-1RA | |
| --- | --- | --- | --- | --- | --- |
| Primary cardiovascular outcomes | No. of matched pairs | N events  (IR/1,000 PY) | N events  (IR/1,000 PY) | HR  (95% CI) | RD/1,000PY  (95% CI) |
| **At least 1 year of follow-up*** |  |  |  |  |  |
| Composite of myocardial infarction or stroke | 31,627 | 266 (10.70) | 259 (11.48) | 0.94 (0.80, 1.12) | -0.78 (-2.69, 1.12) |
| Hospitalization for heart failure | 31,846 | 120 (4.78) | 165 (7.27) | 0.67 (0.53, 0.85) | -2.48 (-3.90, -1.09) |
| MACE outcome^†^ | 11,481 | 181 (19.98) | 210 (23.62) | 0.84 (0.69, 1.02) | -3.64 (-7.98, 0.68) |
| Composite of cardiovascular death or hospitalization for heart failure^†^ | 11,573 | 140 (15.32) | 167 (18.64) | 0.81 (0.65, 1.02) | -3.32 (-7.15, 0.47) |
| Composite of primary outcomes (Medicare only) | 11425 | 249 (27.68) | 288 (32.76) | 0.84 (0.70, 0.99) | -5.08 (-10.21, 0.03) |
| Composite of primary outcomes (All 3 databases) | 31558 | 367 (14.81) | 397 (17.71) | 0.85 (0.74, 0.98) | -2.90 (-5.22, -0.59) |
| **At least 2 years of follow-up^‡^** |  |  |  |  |  |
| Composite of myocardial infarction or stroke | 9313 | 70 (10.33) | 61 (10.68) | 1.00 (0.71, 1.42) | -0.35 (-4.02, 3.25) |
| Hospitalization for heart failure | 9408 | 31 (4.52) | 49 (8.51) | 0.58 (0.37, 0.91) | -3.98 (-6.97, -1.18) |
| MACE outcome^†^ | 3455 | 53 (22.14) | 50 (20.95) | 1.04 (0.71, 1.54) | 1.18 (-7.20, 9.59) |
| Composite of cardiovascular death or hospitalization for heart failure^†^ | 3487 | 35 (14.47) | 49 (20.31) | 0.70 (0.46, 1.08) | -5.84 (-13.45, 1.59) |
| Composite of primary outcomes (Medicare only) | 3430 | 68 (28.68) | 71 (30.27) | 0.93 (0.67, 1.30) | -1.59 (-11.48, 8.26) |
| Composite of primary outcomes (All 3 databases) | 9283 | 96 (14.23) | 97 (17.16) | 0.87 (0.66, 1.16) | -2.92 (-7.44, 1.49) |
| PS: propensity score; IR: Incidence rate; PY: person-years; HR: hazard ratio; CI: confidence intervals; RD: rate difference.  * Analyses were restricted to patients with at least one year of follow-up data from all 3 databases. Follow-up started from one-year post-index until the end of follow-up.  † MACE outcome (which includes hospitalization for myocardial infarction, ischemic or hemorrhagic stroke, or all-cause mortality), and cardiovascular mortality data are only available for the Medicare database.  ‡ Analyses were restricted to patients with at least two years of follow-up data from all 3 databases. Follow-up started from two-year post-index until the end of follow-up. | | | | | |

# Supplemental Table 12. Comparative risk of cardiorenal outcomes among 1:1 PS-matched initiators of empagliflozin vs. liraglutide or dulaglutide

|  | Empagliflozin | GLP-1RA | Empagliflozin vs. GLP-1RA | |
| --- | --- | --- | --- | --- |
| Primary and secondary outcomes | N events  (IR/1,000 PY) | N events  (IR/1,000 PY) | HR  (95% CI) | RD/1,000PY  (95% CI) |
| **Empagliflozin vs. liraglutide (N of matched pairs= 83,274)** | | | | |
| Composite of myocardial infarction or stroke | 881 (13.39) | 813 (13.56) | 1.00 (0.91, 1.10) | -0.17 (-1.45,1.12) |
| Hospitalization for heart failure (HHF) | 384 (5.81) | 490 (8.15) | 0.73 (0.64, 0.84) | -2.34 (-3.27,-1.41) |
| MACE*^†^ | 566 (22.39) | 598 (25.05) | 0.89 (0.80, 1.00) | -2.66 (-5.39,0.06) |
| Composite of cardiovascular mortality or HHF^†^ | 389 (15.31) | 460 (19.21) | 0.80 (0.70, 0.91) | -3.90 (-6.22,-1.58) |
| **Empagliflozin vs. dulaglutide (N of matched pairs= 104,083)** | | | | |
| Composite of myocardial infarction or stroke | 967 (13.38) | 1,057 (14.03) | 0.95 (0.87, 1.03) | -0.65 (-1.85, 0.55) |
| Hospitalization for heart failure | 408 (5.62) | 606 (8.02) | 0.70 (0.62, 0.79) | -2.40 (-3.24, -1.56) |
| MACE*^†^ | 647 (22.67) | 754 (25.42) | 0.89 (0.80, 0.99) | -2.76 (-5.28, -0.24) |
| Composite of cardiovascular mortality or HHF^†^ | 430 (15.00) | 550 (18.48) | 0.82 (0.72, 0.92) | -3.48 (-5.58, -1.39) |
| PS: propensity score; IR: Incidence rate; PY: person-years; HR: hazard ratio; CI: confidence intervals; RD: rate difference.  * MACE includes hospitalizations for myocardial infarction, stroke, or cardiovascular mortality  † Only available in the Medicare database (32,194 1:1 PS-matched pairs for empagliflozin vs liraglutide, and 40,870 1:1 PS-matched pairs for empagliflozin vs dulaglutide) | | | | |
